# Immune Phenotyping of Patients With Acute Vogt-Koyanagi-Harada Syndrome Before and After Glucocorticoids Therapy

**DOI:** 10.3389/fimmu.2021.659150

**Published:** 2021-04-28

**Authors:** Han Jiang, Zhaohui Li, Long Yu, Ying Zhang, Li Zhou, Jianhua Wu, Jing Yuan, Mengyao Han, Tao Xu, Junwen He, Shan Wang, Chengfeng Yu, Sha Pan, Min Wu, Hangyu Liu, Haihong Zeng, Zhu Song, Qiangqiang Wang, Shen Qu, Junwei Zhang, Yafei Huang, Junyan Han

**Affiliations:** ^1^ Department of Immunology, School of Basic Medicine, Tongji Medical College, Huazhong University of Science and Technology, Wuhan, China; ^2^ Retinal and Vitreous Diseases Department of Wuhan Aier Eye Hospital, Wuhan University, Wuhan, China; ^3^ Department of Pathogen Biology, School of Basic Medicine, Tongji Medical College, Huazhong University of Science and Technology, Wuhan, China; ^4^ Ophthalmic Imaging Department of Wuhan Aier Eye Hospital, Wuhan University, Wuhan, China; ^5^ Cataract Department of Wuhan Aier Eye Hospital, Wuhan University, Wuhan, China

**Keywords:** immunopathogenesis, lymphocyte subsets, monocytes, VKH, autoimmunity, cytokine

## Abstract

Previous studies have established that disturbed lymphocytes are involved in the pathogenesis of Vogt-Koyanagi-Harada (VKH) syndrome. Accordingly, glucocorticoids (GCs), with their well-recognized immune-suppressive function, have been widely used for treatment of VKH patients with acute relapses. However, the systemic response of diverse immune cells to GC therapy in VKH is poorly characterized. To address this issue, we analyzed immune cell subpopulations and their phenotype, as well as cytokine profiles in peripheral blood from VKH patients (n=25) and health controls (HCs, n=21) by flow cytometry and luminex technique, respectively. For 16 patients underwent GC therapy (methylprednisolone, MP), the aforementioned measurements as well as the transcriptome data from patients before and after one-week’s GC therapy were also compared to interrogate the systemic immune response to GC therapy. Lymphocyte composition in the blood was different in VKH patients and HCs. VKH patients had significantly higher numbers of T cells with more activated, polarized and differentiated phenotype, more unswitched memory B cells and monocytes, as compared to HCs. MP treatment resulted in decreased frequencies of T cells and NK cells, inhibited NK cell activation and T cell differentiation, and more profoundly, a marked shift in the distribution of monocyte subsets. Collectively, our findings suggest that advanced activation and differentiation, as well as dysregulated numbers of peripheral lymphocytes are the major immunological features of VKH, and GC therapy with MP not only inhibits T cell activation directly, but also affects monocyte subsets, which might combinatorically result in the inhibition of the pathogenic immune response.

## Introduction

Vogt-Koyanagi-Harada syndrome (VKH syndrome) is an acute diffuse uveitis characterized by bilateral, diffuse granulomatous resulted from stromal choroiditis, accompanied by neurologic manifestations consisting of headache and nausea (1). Ocular manifestations of this disease include retinal detachment or subretinal fluid, papilledema and hyperemia of the optic disk at initial onset. The prevalence of VKH varies among different countries and ethnic groups. In Asian, VKH represents one of the most common uveitis entities (2).

The pathogenesis of VKH has been extensively studied during the past decades. It is well established that an autoimmune response against melanocytes is considered to be involved in the pathogenesis of this disease (3). Early studies found that peripheral lymphocytes from patients with VKH could be activated by bovine uveal pigment (4). Subsequently, activated T cells were demonstrated as the predominant cell types in choroidal inflammation, accompanied by the presence of choroidal melanocytes that express HLA-DR, a major histocompatibility complex class II (MHC II) molecule, suggesting activated T cells as the pathogenic cells and the potential role of choroidal melanocytes in antigen presentation and T cell activation (5, 6). Later, different T cell subsets, including cytotoxic T cells (7), T helper (Th) 1 cells (8), and Th17 cells (9), were identified to be pathogenic in the settings of experimental autoimmune uveitis (EAU), a classical animal model for human uveitis, and/or clinical uveitis. In addition to T cells, other immune cells also contribute, including NK cells, B cells and myeloid cells (6). However, the role of these cells in the pathogenesis of VKH is not systemically addressed.

Tissue resident dendritic cells and macrophages are the major professional APCs, while monocytes are regarded as their counterparts in peripheral blood. In addition to antigen presentation (10), monocytes can also release inflammatory cytokines such as interleukin (IL)-6, IL-1β, and TNF-α, thereby directly mediating inflammation. Human monocyte is a heterogeneous cell population that can be classified into three subpopulations based on the differential expression levels of CD14 and CD16 (11). Classical monocytes, which is the major population of human monocytes (~90%), express high levels of CD14 but lack CD16 expression (CD14^++^CD16^−^). The remaining ~10% of human monocytes are further subdivided into the intermediate subset, with low CD16 and high CD14 expression (CD14^++^CD16^+^), and the non-classical subset, with high CD16 but relatively lower CD14 expression (CD14^+^CD16^++^). Classical monocytes express high levels of chemokine receptor CCR2 and low levels of CX3CR1, and perform better in phagocytosis compared to other monocyte subsets, thus contribute to anti-microbial responses (12–14). In contrast to this major subset, human intermediate monocytes express high levels of CCR5, CX3CR1 and HLA-DR, and low levels of CCR2 (13, 15), thus are specialized in antigen processing and presentation. Non-classical monocytes are less characterized, nevertheless, they are demonstrated to have the capacity to patrol blood vessels, resist viruses and stimulate T cell proliferation (16, 17). Although the involvement of these monocyte subsets has been reported in various inflammatory conditions, such as tuberculosis (18), asthma (19), rheumatoid arthritis (RA) (20) and Crohn’s disease (21), their contributions to the pathogenesis of VKH are not fully understood. In addition, a new monocyte subset, CD56^+^ monocyte, was identified and found to be expanded in certain autoimmune diseases such as rheumatoid arthritis and Crohn’s disease (21–23), but their role in VKH remains unclear.

Although numerous new drugs have been developed within the last few decades, the most widely used treatment for acute VKH remains high-dose MP pulse therapy, to which most patients respond well and achieve an amelioration of symptoms within a few days (24, 25). It has been reported that GCs downregulate the expression levels of pro-inflammatory cytokines and adhesion molecules, which are required for cells to pass the blood-brain barrier (BBB). The GC response was reported to be highly cell type-specific in magnitude, and even in terms of the direction of transcriptional regulation (26). It is believed that patients profit most from the direct or indirect dampening effects on T cells by GC treatment, which can inhibit T cell activation, promote apoptosis in immune cells, and additionally exert inhibitory effects on inflammatory mediators such as nitric oxide (NO) (27). However, the effect of GCs on other immune cells including monocytes in VKH is not well characterized. Although it has been demonstrated that *in vitro* treatment of monocytes from healthy donors with GC induced an upregulation of transcripts associated with an anti-inflammatory phenotype, as well as enhanced cell survival, phagocytosis, and chemotaxis (28), the subset-specific response of monocytes to GC treatment and the underlying mechanism in VKH remain to be defined.

This study was designed to systemically characterize and compare the immunological profiles of peripheral blood from patients with VKH and healthy controls. The influence of MP, which has been the most widely prescribed GC in VKH therapy, on immune phenotypes in peripheral blood from VKH patients was also evaluated. Our investigation will offer a better understanding of the function, activation and differentiation status of the leukocyte subpopulations during autoimmune VKH and after GC therapy, and may facilitate a more specific therapeutic regimen for this particular disease, which is a major cause of visual impairment.

## Materials and Methods

### Patients

All patients with uveitis were recruited at the retinal and vitreous diseases department of Wuhan Aier Eye Hospital from April 8, 2017 to December 17, 2020. Healthy controls (HCs) matched for gender and age were recruited among the employees of the hospital. The study protocol was approved by the Ethics Committee of Wuhan Aier Eye Hospital (Clinical Ethical Approval No. 2019IRBKY04). The diagnosis of VKH was based on the newly revised diagnostic criteria of the International Uveitis Conference. Written informed consent was obtained from all patients and HCs before study entry. A venous blood sample was taken from acute VKH patients prior to MP therapy (n=25), after MP therapy for seven consecutive days (n=16), as well as HCs (n=21) (**Tables 1** and **2**). The numbers of subjects used for each assay were indicated in the relevant tables or figures.

**Table 1:** Clinical features of VKH patients and healthy controls.

Clinical features	Acute VKH	Healthy control
**Participants (n)**	25	21
**Age (mean ± SD)**	42.1 ± 12.2	41.4 ± 12.9
**Gender** ** Female**	9 (36.0%)	9 (42.9%)
** Male**	16 (64.0%)	12 (57.1%)
**Subretinal fluid**	18 (72%)	n.a.
**Bullous serous retinal detachment**	2 (8%)	n.a.
**Papilledema**	15 (60.0%)	n.a.
**Hearing loss**	5 (20.0%)	n.a.
**Tinnitus**	4 (16.0%)	n.a.
**Headache**	13 (52.0%)	n.a.

**Table 2:** Clinical features of VKH patients before and after GC treatment.

Clinical features	Before GC	After GC
**Patients (n)**	16	
**Age (mean ± SD)**	44.9 ± 11.8	
**Gender** ** Female**	6 (37.5%)	
** Male**	10 (62.5%)	
**Subretinal fluid**	10 (62.5%)	6 (37.5%)
**Bullous serous retinal detachment**	2 (12.5%)	0
**Papilledema**	8 (50.0%)	5 (31.3%)
**Hearing loss**	4 (25.0%)	0 (0.0%)
**Tinnitus**	3 (18.8%)	0 (0.0%)
**Headache**	10 (62.5%)	0 (0.0%)

### PBMC Isolation

Peripheral blood samples were collected in heparinized vacutainer tubes (BD bioscience, San Jose, CA, USA). After diluted two times by PBS, the blood was added carefully onto the Ficoll-Hypaque layer (3 ml Ficoll-Hypaque per 10 ml blood/PBS mixture), and centrifuged at 800 × g for 30 minutes with brake off, room temperature. Mononuclear layer was obtained and washed twice by PBS. The PBMC aliquots were used immediately for flow cytometry staining, or stocked frozen in fetal calf serum containing 10% DMSO and 5% dextran at -80°C for further test.

### Cell Staining and Flow-Cytometric Analysis

Flow-cytometric analysis was performed according to our standard institutional protocols. All reagents were obtained from BD bioscience unless otherwise indicated. For surface staining, 50 μl freshly collected blood was aliquoted and stained by fluorochrome-conjugated monoclonal antibodies (FCmAbs) against CD45 (HI30), CD14 (M5E2), CD56 (NCAM16.2), CD3 (HIT3a), CD19 (HIB19), CD4 (RPA-T4), CD8 (RPA-T8), HLA-DR (G46-6), CD314 (NKG2D, 1D11), CD25 (M-A251), CD127 (HIL-7R-M21), CCR7 (3D12), CD45RA (HI100), IgD (IA6-2), CD27 (M-T271), CD335 (NKp46, 9E2/NKp46) and CD16 (3G8), to characterize general leukocyte populations and the phenotype of and T, B, NK and monocyte subsets using different staining panels (**Table S1**). After incubated with indicated mAbs for 30 minutes at 4°C in dark, blood samples were lysed by 2 ml red blood cell lysis buffer for 8 minutes, washed twice with 2 ml ice-cold staining buffer (2% FCS and 0.1% sodium azide in 1 × PBS), and then fixed in 300 μl of 1% paraformaldehyde (PFA) for flow cytometric analysis. For intracellular cytokine staining, 5 × 10^5^ PBMCs were stimulated by a cocktail containing phorbol myristate acetate (PMA), ionomycin and brefeldin A for 5 hours in 24-well flat-bottom plates, thereafter the cells were harvested and washed once with PBS. The cells were then stained by fixable viability staining dye (FVS), FCmAbs against CD3 and CD8 for 30 minutes at 4°C in dark, followed by intracellular staining with FCmAbs against IFN-γ (B27), IL-17A (N49-653), and IL-4 (8D4-8) after fixation and permeabilization. Finally, cells were washed twice and re-suspended in 300 μl of 1% PFA. Flow cytometric analysis was performed on a BD Verse instrument and data were analyzed by FlowJo V10 (BD bioscience, San Jose, CA, USA).

### Cytokine Measurements

Blood samples were collected from HCs and VKH patients. After centrifugation, plasma was extracted and stored at −80°C. A human Premixed Multi-Analyte kit (LXSAHM-21 R&D Systems) was used to measure the concentrations of a panel of soluble factors (TNF-α, IL-6, IL-8, CXCL10, IL-10, CCL2, IL-1-β, IFN-γ, CCL20, CCL3, CCL22, IL-4, IL-17, IL-2, IL-13, CXCL9, IL-12, CCL17, IL-21, IL-23, IL-18) in one sample at the same time. Since the concentration of some cytokines from most plasma samples is lower than the detection limit of this assay, as determined by our preliminary experiment, 250 μl plasma is lyophilized and re-suspended in 50 μl PBS (5:1 concentrated). Luminex assays were performed according to the manufacturer’s protocol with standard curves and quality and background controls included. Samples and standards were run in duplicate and mean values were used for data analysis. The concentrations of TGF-β1 in the plasma were assayed using human ELISA kits (Cat# 88-8350, Invitrogen) according to the manufacturer’s instructions.

### RNA Sequencing

Total RNAs of whole blood cells were extracted in accordance with the manual of PAXgene tube (BD bioscience, San Jose, CA, USA). Preparation of library and transcriptomic sequencing were carried out using Illumina Novaseq 6000 (Novogene Bioinformatics Technology Co., Ltd., Beijing, China). Differential expression analysis of two conditions/groups (two biological replicates per condition) was performed using the DESeq2 R package (1.16.1). DESeq2 provide statistical routines for determining differential expression in digital gene expression data using a model based on the negative binomial distribution. The resulting *P* values were adjusted using the Benjamini and Hochberg’s approach for controlling the false discovery rate. Genes with an adjusted *P* value < 0.05 found by DESeq2 were assigned as differentially expressed.

Gene Ontology (GO) enrichment analysis of differentially expressed genes was implemented by the cluster Profiler R package, in which gene length bias was corrected. GO terms with corrected *P* value less than 0.05 were considered significantly enriched by differential expressed genes.

Kyoto Encyclopedia of Genes and Genomes (KEGG) is a database resource for understanding high-level functions and utilities of the biological system, such as the cell, the organism and the ecosystem, from molecular-level information, especially large-scale molecular datasets generated by genome sequencing and other high-throughput experimental technologies (http://www.genome.jp/kegg/). We used cluster Profiler R package to test the statistical enrichment of differential expression genes in KEGG pathways.

### Statistical Analysis

General statistical analysis was performed using GraphPad Prism software. Throughout the study, n refers to the number of subjects where every subject is one data point. Unpaired two-group comparisons were done with Mann–Whitney U-test. Paired group comparisons were done with Wilcoxon test. *P* < 0.05 was considered statistically significant.

## Results

### Dysregulated Peripheral Leukocyte Compartment in Acute VKH Patients

**Figure 1:**
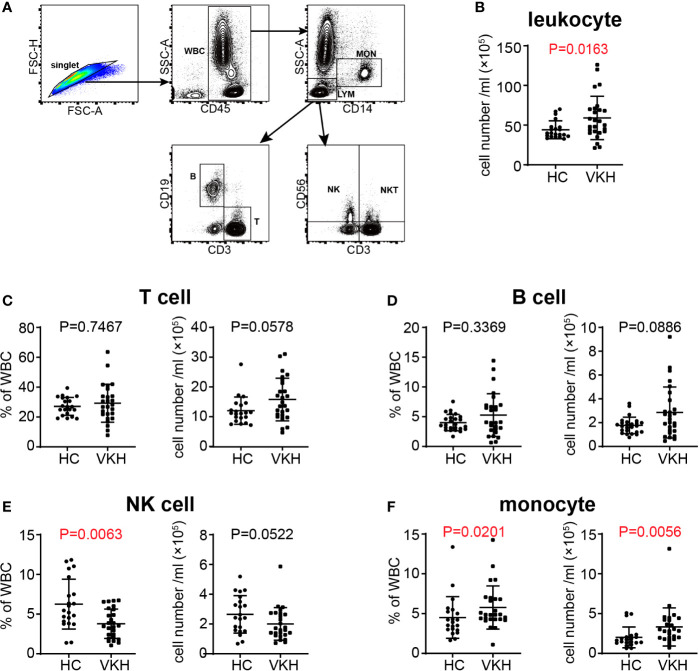
Changes of white blood cells in peripheral blood of VKH patients compared with healthy controls. **(A)** Gating strategy used for the characterization of leukocyte subpopulations. **(B)** The absolute number of leukocytes. **(C)** The absolute number and proportion of T cells. **(D)** the absolute number and proportion of B cells. **(E)** The absolute number and proportion of NK cells. **(F)** the absolute number and proportion of monocytes. Statistical analysis was performed using Mann–Whitney test. WBC, white blood cells; LYM, lymphocytes; MON, monocytes; T, T lymphocytes; B, B lymphocytes; NK, natural killer cells; NKT, natural killer T cells.

To explore immune cell-mediated mechanisms in the development and progression of VKH disease, we examined leukocyte populations in patients with active VKH by flow cytometry (**Figure 1A**). Compared with healthy controls, the number of total leukocytes in peripheral blood of acute VKH patients was increased significantly (**Figure 1B**). There were no significant changes in granulocytes, total lymphocytes and CD3^+^CD56^+^ T cells in terms of both absolute number and relative frequency (**Figures S1A, B**), however, the numbers of T cells and B cells displayed an increased tendency, although this difference failed to reach statistical significance (**Figures 1C, D**). Interestingly, markedly decreased proportion but not absolute number of NK cells was found in VKH patients (*P* = 0.0063) (**Figure 1E**). Finally, both the absolute number and relative frequency of monocytes were significantly increased in VKH patients, as compared to that in HCs (*P* = 0.0201 and *P* = 0.0056, respectively) (**Figure 1F**). Thus, our results in VKH elaborate the proposed notion that T cells and B cells might be pathogenic and NK cells could be protective in most autoimmune diseases, and also raise a question regarding the functional role of increased monocytes.

### Over-Activation of Peripheral T Cells in VKH Patients

**Figure 2:**
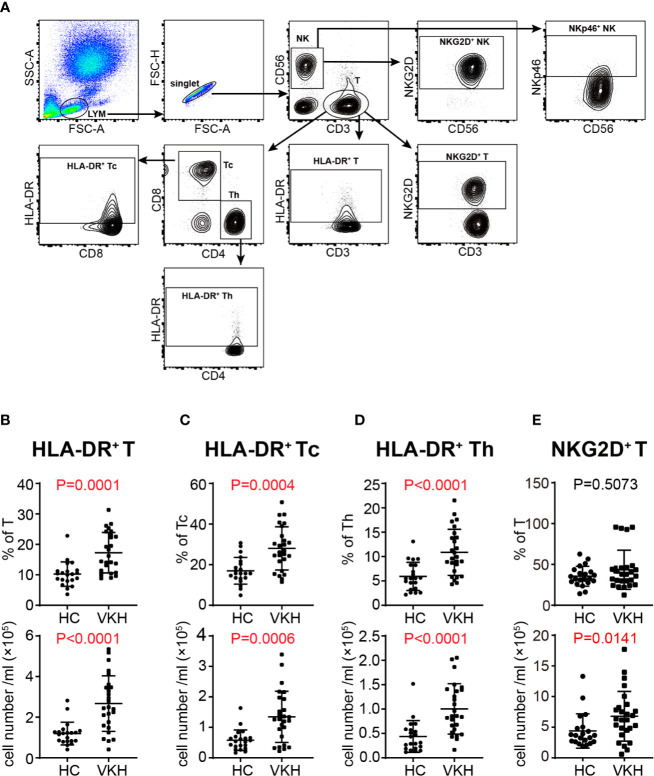
Comparison of activated immune cells between VKH patients and healthy controls. **(A)** Gating strategy used for analysis of activated immune cells. **(B)** The absolute number and proportion of HLA-DR^+^ CD3^+^ T cells. **(C)** The absolute number and proportion of HLA-DR^+^ CD8^+^ T cells. **(D)** The absolute number and proportion of HLA-DR^+^ CD4^+^ T cell. **(E)** The absolute number and proportion of NKG2D^+^ T cells. Statistical analysis was performed using Mann–Whitney test. Th, helper T cells; Tc, cytotoxic T cells.

Activation is a necessary step through which lymphocytes differentiate and exert their function. In this regard, we next determined the activation status of lymphocyte populations by analysis of the expression levels of established T cell and NK cell activation markers HLA-DR, NKG2D and NKp46 (**Figure 2A**). HLA-DR^+^ cytotoxic T lymphocytes (CTLs) have been shown to be increased in patients with HIV infection (29) and in systemic lupus erythematosus (30). In our study, the percentage and absolute number of HLA-DR^+^ T cells in peripheral blood of VKH patients were increased (**Figure 2B**), and these changes were observed in both CD8^+^ and CD4^+^ T cell compartments (**Figures 2C, D**), whereas NKG2D^+^ and NKp46^+^ NK cells did not show much difference (**Figures S2A, B**). NKG2D and NKp46 expression can also be used to reflect the activation status of T cells. In humans, naïve CD8^+^ T cells express NKG2D, whereas CD4^+^ T cells generally do not express NKG2D even after activation, but its expression can be induced under certain pathological conditions, such as Crohn’s disease, juvenile-onset lupus and cytomegalovirus infection (31, 32). In this study, we found that the absolute number of NKG2D^+^ T cells was upregulated (**Figure 2E**), whereas no difference was observed for NKp46^+^ T cells in VKH patients. Together, the elevated expression of HLA-DR and NKG2D in T cells suggested that T cells are over-activated in the context of VKH.

### Advanced Differentiation of T Cells in VKH Patients

**Figure 3:**
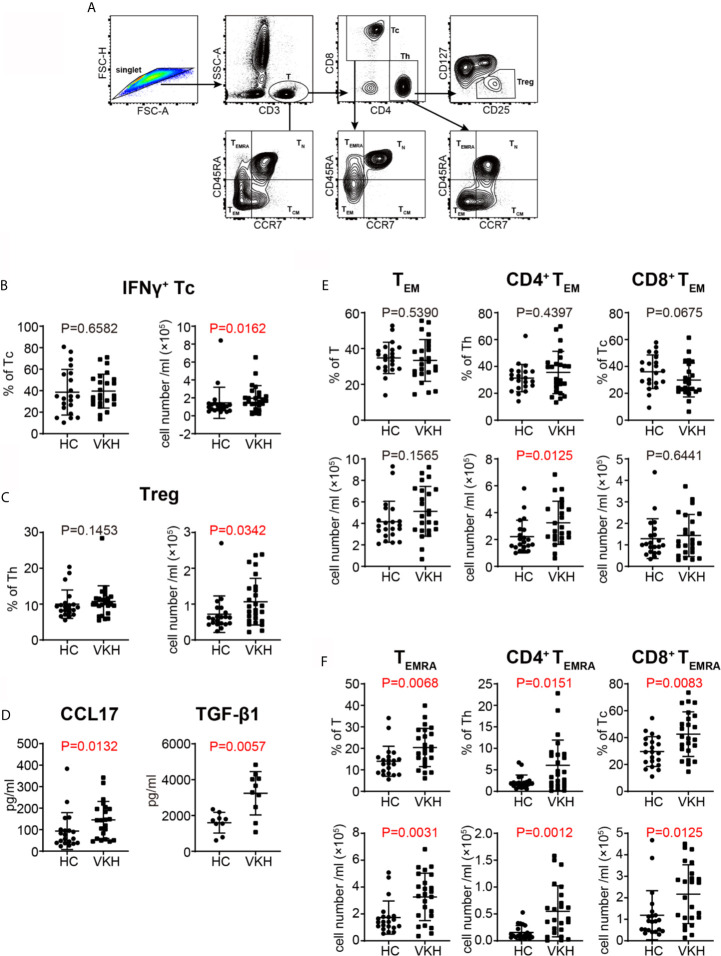
Comparison of T cell subsets defined by cytokine profile and differential status between VKH patients and healthy controls. **(A)** Gating strategy used for T cell classification. **(B)** The absolute number and proportion of IFN-γ^+^ CD8^+^ T cell. **(C)** The absolute number and proportion of Treg. **(D)** plasma levels of CCL17 and TGF-β1 (pg/ml) of HCs and VKH patients. **(E)** The absolute number and proportion of T_EM_ in total T cells, CD4^+^ T and CD8^+^ T cells in HCs and VKH patients. **(F)** The absolute number and proportion of T_EMRA_ in total T cells, CD4^+^ T and CD8^+^ T in HCs and VKH patients. Statistical analysis was performed using Mann–Whitney test. Treg, regulatory T cells; T_N_, naïve T cells; T_CM_, central memory T cells; T_EM_, effector memory T cells; T_EMRA_, CD45RA^+^ effector memory T cells.

Th1 and Th17 cells have been shown to be critical in the pathogenesis of VKH (33, 34). Thus, we next sought to determine the polarization status of peripheral T cells in VKH patients (**Figure 3A**). Compared with healthy controls, VKH patients had significantly increased number of IFN-γ^+^ cytotoxic T cells (Tc), and regulatory T cells (Tregs) (**Figures 3B, C**), whereas Th1, Th2, Th17, IL-4^+^ Tc and IL-17^+^ Tc did not show much difference, in terms of both relative frequency and absolute number (**Figures S3A–E**). Furthermore, after examining the concentration of various cytokines in the plasma of VKH patients and HCs (**Table 3**), we found that CCL17, a chemokine with the potential to recruit Tregs, and TGF-β, a regulatory cytokine mainly secreted by Treg, were also increased in VKH patients (**Figure 3D**).

**Table 3:** Comparison of plasma cytokines between VKH patients and healthy controls.

	VKH (n=22)	HC (n=20)	*P* value
TNF-α	0.73 (0.34-1.64)	0.57 (0.32-1.15)	0.1112
IL-6	0.40 (0.16-1.67)	0.34 (0.16-0.75)	0.6305
IL-8	13.35 (0.12-199.20)	1.97 (0.19-13.14)	0.0002***
CXCL10	21.98 (7.78-124.40)	30.02 (14.94-86.93)	0.1116
IL-10	0.10 (0-3.85)	0.09 (0.01-0.57)	0.9255
CCL2	91.16 (19.43-202.33)	89.61 (49.95-161.87)	0.9752
IL-1β	0.63 (0.38-2.94)	0.61 (0.29-5.47)	0.6218
IFN-γ	3.53 (1.97-22.88)	3.88 (1.63-22.49)	0.9751
CCL20	15.95 (1.85-85.75)	14.64 (5.60-204.19)	0.8273
CCL3	14.73 (5.90-44.47)	8.00 (4.60-49.30)	0.0621
CCL22	94.13 (9.30-284.07)	95.29 (56.21-259.00)	0.9305
IL-4	4.33 (0.11-21.70)	4.79 (3.83-7.75)	0.0936
IL-17A	0.54 (0.35-5.24)	0.47 (0.24-1.32)	0.5343
IL-2	1.15 (0.84-4.34)	1.06 (0.69-1.66)	0.0604
IL-13	16.80 (9.70-48.79)	16.43 (5.14-27.31)	0.5860
CXCL9	49.52 (39.09-129.64)	47.58 (36.46-111.30)	0.5279
IL-12	3.58 (2.33-4.71)	4.14 (2.94-7.32)	0.0599
CCL17	144.12 (44.85-342.22)	67.19 (23.16-383.50)	0.0132*
IL-21	1.72 (1.05-8.52)	1.85 (1.00-8.52)	0.7884
IL-23	25.37 (5.90-136.11)	34.25 (7.41-88.04)	0.2518
IL-18	83.20 (36.80-152.98)	64.29 (5.57-166.69)	0.2171

Cytokine concentrations are expressed in pg/ml as median (min-max). Data were analyzed by Mann–Whitney test; *P < 0.05; **P < 0.01; ***P < 0.001; ****P < 0.0001.

CCR7 and CD45RA have been widely used to define the differentiation status of T cells (35). According to this paradigm, naïve T cells (T_N_) express both CCR7 and CD45RA, central memory T cells (T_CM_) express CCR7 but not CD45RA, while effector memory T cells (T_EM_) lack the expression of both CCR7 or CD45RA. In humans, there is a third memory T cell subset, T_EMRA_ cell, which expresses CD45RA but lack CCR7 expression. Further analysis of T cell subpopulations demonstrated that certain differentiated subsets were variably affected in VKH patients. Although there were no significant changes in naïve T cells and T_CM_ cells in terms of both absolute number and relative frequency (**Figures S3F, G**), the number of CD4^+^ T_EM_ cells was increased (**Figure 3E**), and the frequencies of T_EMRA_ cells were also increased in both CD8^+^ and CD4^+^ T cell compartments in VKH patients (**Figure 3F**). In sum, these results demonstrated that VKH patients harbor more polarized IFN-γ^+^ Tc cells, as well as more differentiated T_EM_ and T_EMRA_ cells in peripheral blood, suggesting the pathogenic role of these cells. However, the clinical meaning of increased Tregs and Treg-related cytokines in VKH patients requires further investigation.

### Increased Number of Unswitched Memory B Cells in VKH Patients

**Figure 4:**
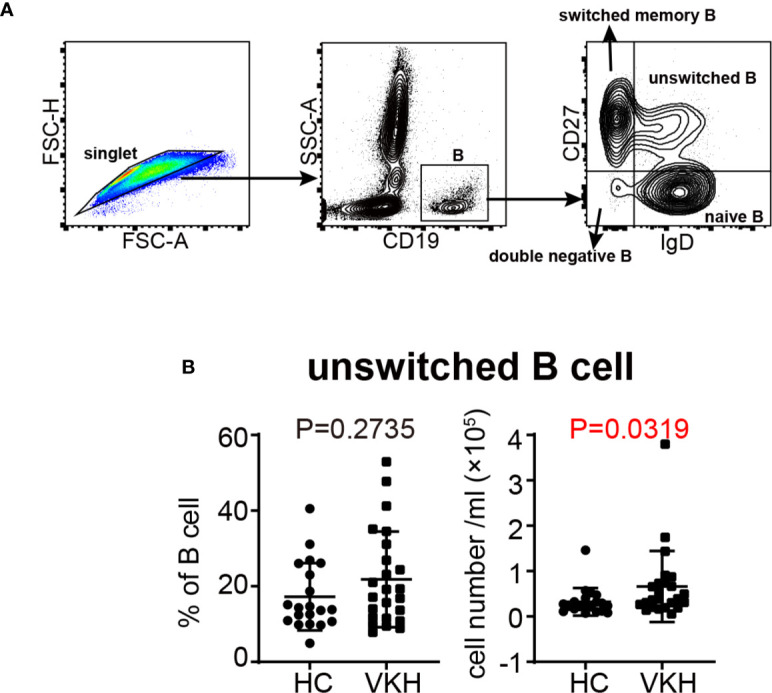
Comparison of B cell subsets between VKH patients and healthy controls. **(A)** Gating strategy used for the characterization of B cell subsets. **(B)** The absolute number and proportion of unswitched B cells. Statistical analysis was performed using Mann–Whitney test.

Since we observed an increased tendency of B cells in VKH patients (**Figure 1D**), and because of the heterogeneity of B cells, we next examined B cell subsets by flow cytometry (**Figure 4A**), to determine the specific B cell subsets that may play an important role in VKH. There are four relatively well-characterized CD19^+^ B cell subpopulations in human peripheral blood, naïve (CD27^-^IgD^+^), unswitched memory (CD27^+^IgD^+^), switched memory (CD27^+^IgD^-^) and double negative late memory (CD27^-^IgD^-^) B cells (36). As demonstrated in **Figure 4B**, the number of unswitched memory B cells was markedly increased in VKH patients. The numbers of naïve B cells, switched memory B cells and late memory B cells tended to be increased as well, but these differences failed to reach statistical significance (**Figures S4A–C**). Our findings thus suggest the pathogenic role of B cells, especially unswitched memory B cells, in VKH.

### Alteration of Monocyte Subsets in VKH Patients

**Figure 5:**
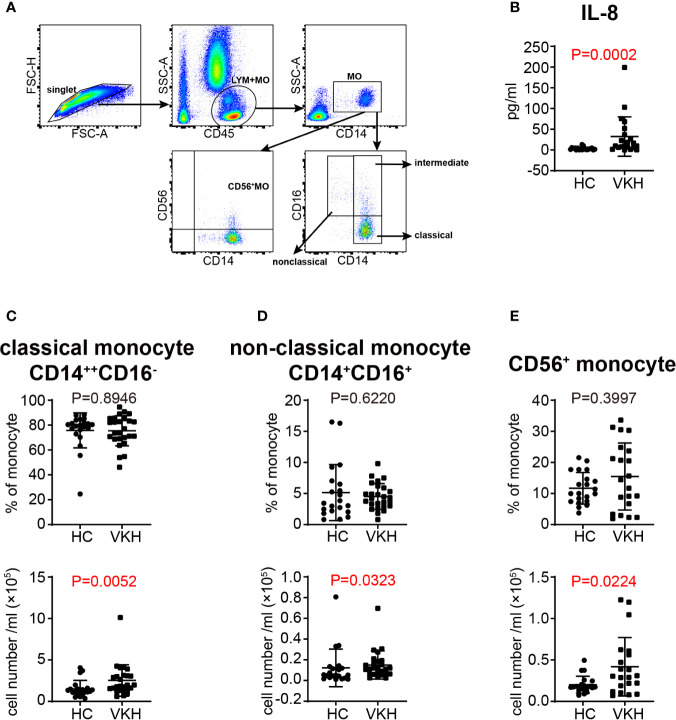
Changes of monocyte subsets in VKH patients compared with healthy controls. **(A)** Gating strategy used for the characterization of monocyte subsets. **(B)** Plasma level of IL-8 (pg/ml) of HCs and VKH patients. The absolute number and proportion of **(C)** classical monocytes, **(D)** non-classical monocytes and **(E)** CD56^+^ monocytes. Statistical analysis was performed using Mann–Whitney test.

Monocytes have been considered as the precursors of tissue macrophages and monocyte derived DC in periphery. Increased number of monocytes was noted in VKH patients (**Figure 1C**). Therefore, we went on to examine the changes of monocyte subsets in the peripheral blood (**Figure 5A**) and concentration of cytokines related to monocyte in the plasma of VKH patients. We found that IL-8, which could be secreted by monocytes and other cells in the peripheral blood, was increased in VKH patients (**Figure 5B**). This result is consistent with the increased number of monocytes in VKH patients. Moreover, the numbers of classical, non-classical and CD56^+^ monocytes were all significantly increased in VKH patients as compared to HCs (**Figures 5C–E**). However, both the proportion and number of intermediate monocytes were not statistically different between these two groups (**Figure S4D**).

### GC Treatment Influences White Blood Cells in VKH Patients

**Figure 6:**
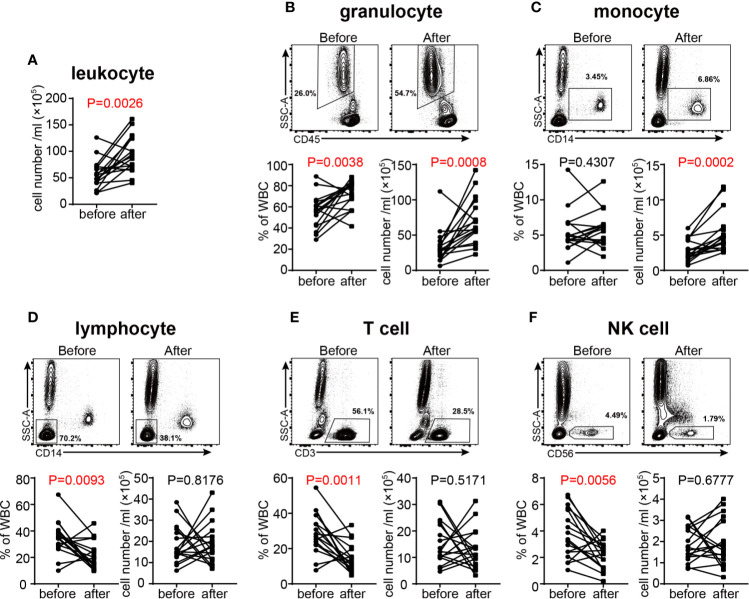
Changes of white blood cells in acute VKH patients before and after GC treatment (n = 16). **(A)** Changes in the total number of white blood cells. The absolute number and proportion of granulocytes **(B)**, monocytes **(C)**, lymphocytes **(D)**, T cells **(E)** and NK cells **(F)**. Statistical analysis was performed using Wilcoxon test.

Previous studies have found that treating healthy subjects with high dose of GC (400 mg) resulted in transient neutrophil demargination and a decrease in circulating lymphocytes peaking at 4 hours post treatment (37). In our study, we examined the changes of leukocyte subpopulations in VKH patients after GC therapy for seven consecutive days. The total number of white blood cells in peripheral blood of patients was increased after GC therapy (**Figure 6A**), which was mainly ascribed to the increase of granulocytes, the most abundant leukocytes in human peripheral blood (**Figure 6B**). The average number of total monocytes was increased from 3 × 10^5^ to 5 × 10^5^ after GC treatment (**Figure 6C**). In contrast, the number of total lymphocytes did not change significantly, whereas the percentage of lymphocytes decreased after treatment (**Figure 6D**). When lymphocyte subsets were compared, the proportion of T cells and NK cells were decreased (**Figures 6E, F**), whereas B cells and NKT cells did not change significantly in response to GC treatment (**Figures S5A, B**). In together, our results indicated that GC treatment resulted in increased numbers of monocytes and granulocytes, but decreased proportions of T cells and NK cells in VKH patients.

### GC Treatment Inhibits the Activation of NK Cells and the Differentiation of T Cells

**Figure 7:**
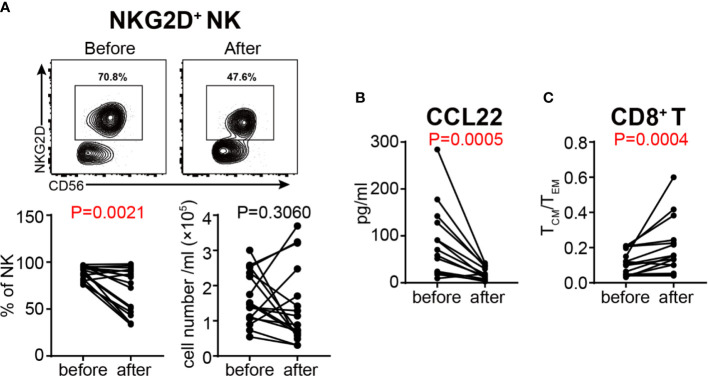
Assessment of the activation status of NK cells and the differentiation status of T cells in VKH patients before and after GC treatment. **(A)** The absolute number and proportion of NKG2D^+^ NK cells. **(B)** plasma level of CCL22 (pg/ml) in HCs and VKH patients. **(C)** The T_CM_/T_EM_ ratio of CD8^+^ T cells. Statistical analysis was performed using Wilcoxon test.

In order to determine the effects of GC therapy on cell activation, we next analyzed the activation and inhibition markers on the surface of T cells and NK cells. Although NKp46^+^ NK, HLA-DR^+^ T, HLA-DR^+^ Th, HLA-DR^+^ Tc, NKG2D^+^ T, NKp46^+^ T and Treg cells did not show any significant changes (**Figures S6A–G**), the percentage of NKG2D^+^ NK cells was decreased significantly after GC therapy (**Figure 7A**), indicating that GC therapy inhibits the activation of NK cells. We also compared the content of various cytokines in the plasma of VKH patients before and after GC therapy (**Table 4**), and found that CCL22, one of the chemokines with the potential to recruit Tregs, was decreased after GC treatment (**Figure 7B**). Next, we focused on cytokine profile-defined T cell subsets, GC treatment did not change both the absolute numbers and relative frequencies of Th1, Th2, Th17, IFN-γ^+^ Tc, IL-4^+^ Tc and IL-17^+^ Tc subsets (**Figures S7A–F**). When T cell differentiation status was examined, the relative frequencies of CD4^+^ T_N_, T_CM_ (within both CD4^+^ and CD8^+^ T cell compartments) and CD8^+^ T_EM_ cells changed significantly after GC therapy (**Figures S8A–C**). T_CM_/T_EM_ ratio has been used to reflect the differentiation status of T cells (38), We therefore compared this parameter before and after GC treatment and found a highly significant increase (*P* = 0.0004) of the T_CM_/T_EM_ ratio among CD8^+^ T cells after GC treatment (**Figure 7C**).

**Table 4:** Comparison of plasma cytokines in VKH patients before and after GC treatment.

	Before GC (n=13)	After GC (n=13)	*P* value
TNF-α	0.61 (0.32-6.85)	0.57 (0.32-1.34)	0.1099
IL-6	0.35 (0.16-2.48)	0.30 (0.16-1.29)	0.1465
IL-8	11.65 (0.12-243.18)	11.65 (0.41-243.18)	0.9460
CXCL10	27.95 (4.98-151.14)	16.00 (4.98-151.14)	0.0681
IL-10	0.13 (0-74.61)	0.06 (0-2.81)	0.0425*
CCL2	85.77 (19.43-161.87)	57.79 (39.28-129.49)	0.0398*
IL-1β	0.61 (0.29-5.86)	0.52 (0.37-5.86)	0.5879
IFN-γ	3.88 (1.63-51.26)	3.16 (1.86-5.67)	0.2925
CCL20	14.64 (1.85-204.19)	7.37 (1.85-31.15)	0.4973
CCL3	11.26 (4.60-59.54)	11.26 (4.60-59.54)	0.6221
CCL22	41.98 (2.83-259.00)	18.74 (2.83-41.98)	0.0005***
IL-4	5.13 (0.11-10.55)	5.13 (2.10-10.55)	0.3850
IL-17A	0.54 (0.22-5.81)	0.54 (0.22-5.81)	0.3481
IL-2	1.21 (0.69-6.40)	1.21 (0.81-6.40)	0.8145
IL-13	17.56 (5.14-69.60)	17.56 (9.70-39.72)	0.7646
CXCL9	48.88 (36.46-264.50)	44.33 (39.09-71.09)	0.3613
IL-12	4.14 (2.33-42.03)	3.36 (2.53-28.36)	0.8066
CCL17	64.32 (23.16-383.50)	64.32 (30.22-221.36)	0.3757
IL-21	1.85 (1.00-20.10)	1.39 (1.00-3.45)	0.1851
IL-23	34.25 (3.94-88.04)	10.83 (3.94-83.63)	0.0681
IL-18	76.99 (5.57-215.64)	76.99 (27.02-215.64)	0.3804

Cytokine concentrations are expressed in pg/ml as median (min-max). Data were analyzed by Wilcoxon test; *P < 0.05; ***P < 0.001.

### GC Treatment Affects the Distribution of Monocyte Subsets in VKH Patients

**Figure 8:**
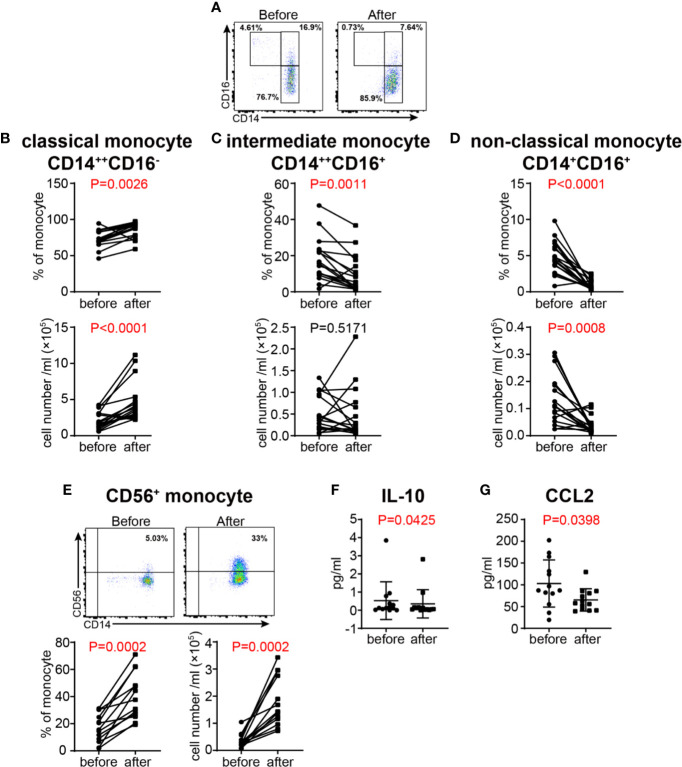
Changes of monocyte subsets in VKH patients before and after GC treatment. **(A)** Monocyte subsets in a VKH patient before and after GC treatment. The absolute number and proportion of classical monocytes **(B)**, intermediate monocytes **(C)**, non-classical monocytes **(D)** and **(E)** CD56^+^ monocytes. Statistical analysis was performed using Wilcoxon test. Plasma levels of IL-10 **(F)** andCCL2 (pg/ml) **(G)** in VKH patients before and after GC treatment.

After GC treatment, the proportions of the three monocyte subsets defined by the expression of CD14 and CD16 were significantly altered (**Figure 8A**). Both the proportion and absolute number of CD14^++^CD16^−^ classical subset were increased (**Figure 8B**), whereas the proportion of CD14^++^CD16^+^ intermediate subset, and the proportion and absolute number of CD14^+^CD16^+^ non-classical subset were decreased (**Figures 8C, D**). Interestingly, the newly defined CD14^+^CD56^+^ monocyte subset were significantly increased after GC treatment in terms of both relative frequency and absolute number (**Figure 8E**), indicating that this monocyte subset might play a role in the remission of VKH. Additionally, we examined the concentration of cytokines related to monocyte in the plasma of VKH patients before and after GC treatment, and found that CCL2, a chemokine with the potential to recruit monocyte and T cell to the sites of inflammation induced by either tissue injury or infection (39), were also decreased after GC treatment. In addition, IL-10, a regulatory cytokine secreted by monocyte and Treg, was decreased after GC treatment as well, however, plasma IL-10 levels were extremely low in both groups and the biological meaning of this difference is questionable (**Figures 8F, G**). Therefore, whether IL-10 and CCL2 are involved in the function of CD14^+^CD56^+^ monocytes in GC treatment of VKH patients warrants further investigations.

### GC Treatment Affects the Proliferation, Activation, Differentiation and Migration of Immune Cells at the Transcriptomic Level

**Figure 9:**
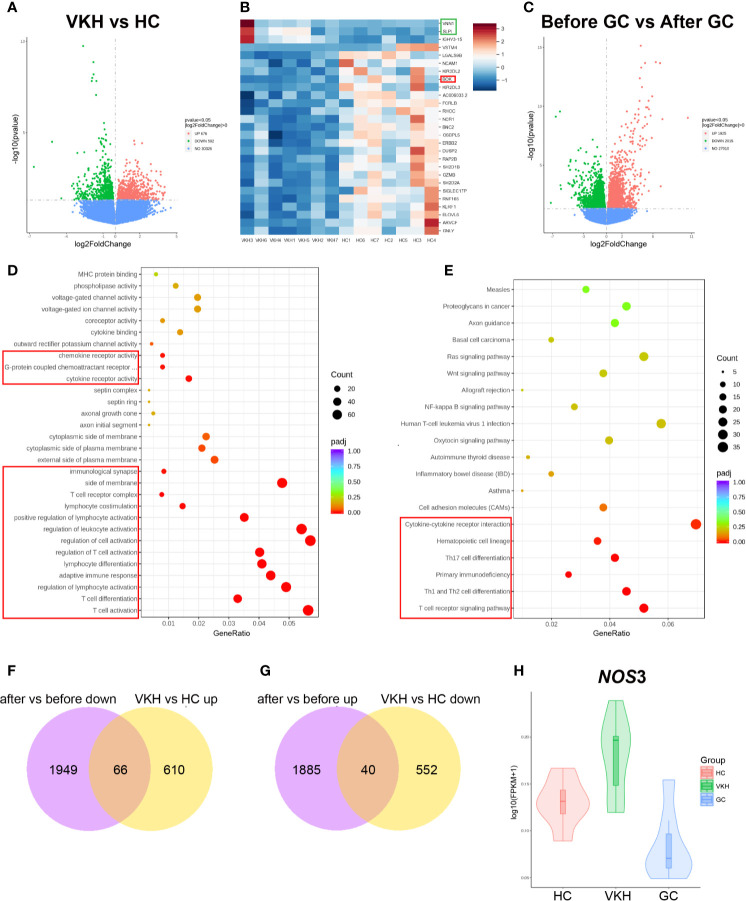
Gene expression in the peripheral blood from VKH patients before (before, n=7) and after (after, n=7) GC treatment for one week, as well as healthy controls (HC, n=7). **(A)** Volcano plots illustrating the differential RNA expression levels between VKH patients and healthy controls. **(B)** Heatmap shows differentially expressed genes in VKH patients and HCs. **(C)** Volcano plots illustrating the differential RNA expression levels between patients before and after GC treatment. Functional enrichment analysis revealing differentially expressed genes in VKH patients before and after GC by KEGG **(D)** and GO enrichment **(E)**. **(F)** Genes up-regulated in VKH and down-regulated after GC therapy. **(G)** Genes down-regulated in VKH and up-regulated after GC therapy. **(H)** Violin plot of *NOS*3 expression in HCs, VKH patients before and after GC treatment.

To further interrogate the effects of GC treatment on peripheral immune cells in the context of VKH, we analyzed transcriptomic changes in peripheral blood from healthy controls and VKH patients before and after GC treatment. For this purpose, peripheral blood samples were collected from HCs, as well as patients before and after GC treatment for one week (seven samples per group). Transcriptomic profiles of these samples were examined by RNA sequencing. Our RNA-seq analysis revealed 1268 differentially expressed genes (DEGs), of which 676 genes were upregulated and 592 genes were downregulated in VKH patients compared to healthy controls (*P* < 0.05) (**Figure 9A**). NK cell-related genes in VKH patients were significantly downregulated, which is in agreement with our finding by flow cytometry that the percentage of CD56^+^ NK cells was significantly reduced in the VKH patients (**Figure 1E**). However, genes that are associated with NK cell activation (e.g., *SH2D1B* and *KLRF1*) and inhibition (e.g., *KIR2DL3* and *KIR3DL2*) were both reduced, suggesting the complex role of NK cells in the pathogenesis of VKH diseases. In addition, *BOK*, the gene related to apoptosis, was down-regulated, and genes related to inflammation (e.g., *SLPI* and *VNN1*) were upregulated in VKH patients (**Figure 9B**).

Differential gene expression analysis of VKH patients before and after GC treatment revealed that 1925 genes were upregulated and 2015 genes were downregulated with P value less than 0.05 after GC treatment (**Figure 9C**). GO enrichment analysis showed that genes associated with lymphocyte (especially T cell) activation and differentiation were downregulated (**Figure 9D**). KEGG enrichment analysis of the differential genes revealed that gene modules associated with Th1, Th2 and Th17 cell differentiation, as well as ‘chemokines and chemokine receptor interaction’ were all downregulated (**Figure 9E**). Therefore, these results indicated that GC treatment might counteract VKH disease through inhibiting T cell activation, differentiation and cytokine/chemokine interaction, which is consistent with our results that the total number of T cells, the number and proportion of activated T cells were increased in the peripheral blood of VKH patients (**Figure 1C** and **Figures 2A–E**). GO pathway analysis also revealed that genes associated with granulocyte activation and degranulation were mostly upregulated after GC therapy, which is consistent with the increased number of granulocytes after GC therapy found by flow cytometry (**Figure S9A**). In addition, KEGG analysis indicated that pathways associated with apoptosis were upregulated after GC therapy (**Figure S9B**). Together, these results demonstrated that GC treatment of VKH may function through inhibiting the activation, proliferation and differentiation, and promoting the apoptosis of immune cells.

In order to identify the key genes that contribute to both the occurrence of VKH and the effects of GC treatment, we performed integrated analysis of DEGs in all three groups. We found that 66 genes were upregulated in VKH patients but downregulated after GC therapy (**Figure 9F**), and 40 genes were downregulated in VKH patients but upregulated after GC therapy (**Figure 9G**). Among these 106 genes, a key gene *NOS3* (Nitric oxide synthase), which plays crucial roles in regulating vascular tone, cellular proliferation, leukocyte adhesion and platelet aggregation through production of NO in the vascular endothelium (40), was elevated in VKH patients and can be reduced after GC therapy (**Figure 9H**), suggesting the dysregulated inflammatory response in VKH patients, and the anti-inflammatory effect of GC treatment in VKH.

## Discussion

VKH is a complex disease involving multiple interactions between different immune cell populations. Although much effort has been made in elucidating the pathogenesis of VKH (3), the role of different leukocytes in this disease remains incompletely understood. In addition, despite the widely use of GCs in clinical management of VKH, the specific anti-inflammatory effects of GCs (i.e. MP) on different cellular compartments in VKH disease are yet to be defined. In this observational study, we performed a comprehensive analysis of the peripheral immune system in VKH patients before and after GC treatment. Our analysis included the subsets of T lymphocytes, NK cells, B cells, monocytes and granulocytes, as well as the functional potential of these cell subsets. The major findings of this study are presented as follows: Firstly, VKH patients harbor a dysregulated lymphocyte compartment in peripheral blood, including higher numbers of T cells, unswitched memory B cells and monocytes, as compared to healthy controls. Secondly, peripheral T cells from VKH patients are more activated, polarized and differentiated than those from HCs. Thirdly, leukocytes from VKH patients are generally more inflammatory and less prone to apoptosis than those from HCs, as indicated by transcriptomic analysis. Finally, GC treatment is not only able to inhibit the activation of NK cells and the differentiation of T cells, but also capable of polarizing monocytes toward an inhibitory phenotype, thereby counteracting the pathogenic immune response in VKH.

Dysregulated T cell compartment has been well-recognized as a prominent feature of multiple autoimmune diseases including VKH. Increased numbers of activated T lymphocytes have been reported in patients with active uveitis (41, 42), and polarized Th1 and/or Th17 lymphocytes are thought to play a major role in the pathogenesis of VKH (9, 33, 34). In our study, total T cells are elevated in VKH patients, and the percentages of CD4^+^HLA-DR^+^, CD8^+^HLA-DR^+^ and NKG2D^+^ T cells are significantly higher in VKH patients than that in HCs, elaborating previous findings that activated T cells play a pathogenic role in VKH. With regard to T cell polarization, the number of Th1 cells in VKH patients is only slightly higher than in controls, and the difference was not significant. However, we do notice an increase in the number of IFN-γ-secreting CD8^+^ T cells, suggesting that T cells from patients with VKH disease are apt to produce IFN-γ, thereby promoting inflammation. Upon antigen challenge, naïve T cells further differentiate into central memory T cells (T_CM_), which home to T cell areas of secondary lymphoid organs and have little effector function, but are able to readily proliferate and differentiate to effector cells in response to antigenic stimulation, and effector memory T cells (T_EM_) that migrate to inflamed peripheral tissues and display immediate effector function. Another memory T cell subset in human, T_EMRA_ cells, which express CD45RA but lack CCR7 expression, is also thought to be able to exert rapid effector function (43). Our results demonstrated a significant increase in the percentage of T_EMRA_ and CD4^+^ T_EM_ cells in VKH patients. Furthermore, after GC treatment, we found that CD8^+^ T_CM_ was increased while CD8^+^ T_EM_ was decreased, which resulted in an increased ratio of T_CM_/T_EM_, suggesting that T cells are more differentiated in VKH, and switch to a relatively static memory state from an activated state upon GC treatment.

Regarding the changes of peripheral Tregs (e.g., number, frequency and functionality) in autoimmunity, conflicting results have been reported in a variety of autoimmune diseases, including type 1 diabetes, multiple sclerosis, systemic lupus erythematosus (SLE), myasthenia gravis, rheumatoid arthritis (RA) (44–47). In this study, we observed that the number of CD4^+^CD127^-^CD25^++^ Tregs was increased, accompanied by elevated plasma levels of chemokines and cytokines that recruit and induce differentiation of Tregs, such as CCL17 and TGF-β. Moreover, CCL22, another chemokine with the potential to recruit Tregs, was decreased after GC treatment. The increase of Tregs in VKH is somewhat counterintuitive given the well-established protective role of these cells in autoimmunity. Several interpretations could be applied to these results: Firstly, the number of peripheral Tregs is simply not reflective of that of tissue Tregs, which are more related to autoimmunity. Secondly, in addition to CD4, CD127 and CD25, other markers are needed to identify functional subsets of Tregs, especially those autoimmune-protective Treg subsets. Finally, the increased number of these cells in VKH patients may simply reflect a compensatory response of our immune system in an effort to maintain immune homeostasis but unfortunately fail to control the inflammatory response (48), either due to the still limited numbers or their potential functional impairment. In support of the latter notion, recent reports have demonstrated that APCs from patients with autoimmune diseases (such as RA) could inhibit the function of Tregs in their peripheral blood, suggesting that APCs (e.g., monocytes, will discuss later) may be critical for secondary Treg cell dysfunction in autoimmune patients (49).

B lymphocytes could contribute to the pathogenesis of autoimmune diseases by producing autoantibodies and/or through acting as APCs (50). B cell infiltration was found in uveal tissues obtained from two patients with VKH, indicating its involvement in VKH (51). Here we found that peripheral B cells were increased in VKH patients, further support the pathogenic role of B cells in VKH. Moreover, among all B cell subsets, IgD^+^CD27^+^ B cell was the most increased subset. As a typical memory B cell subset, IgD^+^CD27^+^ B cell has key features of enhanced responsiveness, metabolism, proliferation, as well as a propensity to differentiate into plasmablast, together arguing for these cells being antigen-experienced. In various functional activation assays, the IgD^+^CD27^+^ B cells showed a higher and faster reactivity than naïve B cells (52). Thus, the increase of IgD^+^CD27^+^ memory B cells may suggest a prominent role of memory B cells in the pathogenesis of VKH. However, we did not find significant changes of B cell subsets after GC treatment (**Figures S10A–E**). It’s likely that GC treatment may not be able to counteract the dysregulation of B cell subsets, although B cells express the glucocorticoid receptor throughout development and GC therapy has been effective in treating autoimmune diseases in which antibody contributes to pathology, such as rheumatoid arthritis (53).

With both regulatory and promoting effects, NK cells seem to act as a two-edged weapon in autoimmune disorders. On the one hand, decreased NK cell frequency and impaired NK cell cytotoxicity were observed in a variety of autoimmune diseases (e.g., systemic juvenile rheumatoid arthritis, SLE, psoriasis and Graves’ disease), implying a protective role of NK cells in controlling autoimmunity (54, 55). NK cells may limit autoimmune responses by inhibiting the proliferation and activation of auto-reactive T lymphocytes, and hampering the activation of monocytes. Accordingly, deficiency or decreased NK cell activity might result in sustained activation of auto-reactive T cell and increased monocytes, thus aggravate inflammatory responses and tissue injury. On the other hand, NK cells also have deleterious roles in the pathogenesis of autoimmune diseases. Increasing evidence has demonstrated that NK cells may participate in nearly all steps in the pathogenesis of autoimmunity, including direct killing, leading to the release of self-antigens, priming T cell activation at the initial and late stages, and finally resulting in tissue destruction (56). In this study, the proportion of NK cells was decreased, accompanied by the downregulation of NK cell-related genes in VKH patients, supporting the overall regulatory role of NK cells in VKH, and the decrease of these cells may result in sustained activation of autoimmune T cells. However, NKG2D^+^ NK cells were decreased after GC treatment, suggesting the complex role of NK cells in VKH. NKG2D^+^ NK cells may represent a pro-inflammatory NK cell subset, which could be eliminated by GC treatment.

It has been well-established that GCs increase bone marrow-derived granulocytes in the blood stream. This effect is utilized clinically to combat granulocytopenia, usually in combination with G-CSF (57). In this study, GC treatment resulted in elevated neutrophils in VKH patients, which is in agreement with previous reports. The increased number of peripheral neutrophils in response to GC treatment could result from the release of these cells from marginal pool to circulation. In fact, it has been reported that GCs could suppress cell adhesion *via* reducing L-selectin expression in PMNs and E-and P-selectin in endothelial cells (58, 59). This notion is further supported by our transcriptomic analysis in which the upregulated expression of genes related to granulocytes, and the decreased expression of cell adhesion molecules were simultaneously observed following GC treatment. Similar mechanisms could also be used to explain the changes of blood monocytes in VKH patients after GC treatment.

Monocytes are rapidly mobilized in large numbers to inflamed sites, where they exert both T cell-dependent function through antigen presentation, and T cell-independent effects such as phagocytic activity as well as secretion of pro-inflammatory cytokines and chemokines. The three major monocyte subsets had differential functional properties (12). Classical monocytes are particularly potent in phagocytosis and resisting bacterial infection, intermediate monocytes are more capable of antigen presentation and stimulating T cell differentiation, whereas non-classical monocytes are specialized in patrolling blood vessels, resisting viruses and stimulating T cell proliferation (12, 60). In contrast to these three major monocyte subsets, the function of the newly identified human CD14^+^CD56^+^ monocytes remains undefined, despite increased number of these cells found in certain pathological conditions, such as Crohn’s disease and rheumatoid arthritis (21, 23). In this study, peripheral monocytes, especially classical monocytes and CD56^+^ monocytes were expanded in VKH patients. Intermediate and non-classical monocytes are also showing a tendency to increase in VKH patients. These findings suggest that the overall effect of peripheral monocytes in VKH patients may be pro-inflammatory. Furthermore, IL-8, a chemokine produced by multiple cell types including peripheral monocytes, was upregulated in VKH patients. IL-8 is the primary cytokine involved in the recruitment of neutrophils to the site of damage or infection and plays important roles in the ocular inflammation and inducing ocular angiogenesis in any parts of the eyes (61). This result suggests that IL-8 may be responsible for the overall pro-inflammatory effect of monocytes in VKH.

Widely used in clinic practice to contain inflammation-related diseases, GCs are well-recognized as an anti-inflammatory agent. With regard to monocytes, GC treatment not just suppresses the pro-inflammatory properties of monocytes but rather induces a distinct functional phenotype. Transcriptomic analysis found that GC-treated monocytes are able to limit tissue damage by improving the anti-oxidative properties and increasing the capacity for phagocytosis in response to pro-inflammatory stimuli (e.g., microbial agents, particles, and cellular debris) (62). Our data elaborate this finding at the cellular level, indicating that GC treatment results in significantly increased number of classical monocytes that have strong phagocytic capacity, accompanied by the decreased proportion of intermediate and non-classical monocytes which possess stronger antigen presentation capacity. These results are in agreement with previous *in vivo* studies in healthy individuals and patients with certain autoimmune disorders (63), but are contradictory to the findings by the Nussenblatt group, which showed that the proportion of intermediate monocytes are increased in uveitis patients received GC therapy, and after *in vitro* treatment of purified monocytes with dexamethasone (64). However, the *in vivo* short-term effect of GCs on monocyte subsets observed by us and others may be different from the long-term effect of GCs described by the Nussenblatt group. In addition, *in vitro* treatment of purified monocyte or monocyte subsets with GCs may not faithfully recapitulate what happened *in vivo*, in which multiple cell types and cell-cell interactions are all subject to the regulation of GCs, and the combined effects could be fundamentally different. Our finding that GC treatment resulted in a further increase of the already expanded CD56^+^ monocytes in VKH patients is not reported in the literature, to the best of our knowledge. It is possible that these cells are regulatory and are increased in VKH patients in an effort to inhibit exaggerated inflammatory response, while in response to GC treatment, these cells quickly improve their quantity and quality, thereby contributing to the successful control of VKH. However, the exact functional properties of CD56^+^ monocytes and underlying mechanisms of their response to GC treatment in VKH require further investigations.

*NOS3* is one of the several forms of nitric oxide synthase, which catalyzes the formation of nitric oxide (NO). It has been already known that NO is synthesized by many cell types that are involved in immune responses and inflammatory reactions (40), but the role of NO in immune diseases and inflammation is still unclear. It has been reported that the increased serum nitrite (NO surrogate) in patients suffering from active RA is associated with disease activity, inflammatory cytokines, and endothelial function (65). As demonstrated by our RNA-Seq results, *NOS3* was increased in VKH patients, but decreased after GC treatment, indicating that the concentration of nitrite in peripheral blood may be related to VKH activity, and the measurement of nitrite could be a diagnostic and prognostic candidate for the management of VKH patients.

In conclusion, the comprehensive analysis of blood leukocytes from VKH patients and HCs described in this study reveals a perturbation of the lymphocyte compartment in VKH, exemplified by the increased activation and proliferation of T cells in VKH patients. GC therapy not only affects the proliferation and apoptosis of lymphocytes, especially T cells, but also affects the distribution of monocyte subsets. Our results also indicate that CD56^+^ monocytes may be involved in the remission of inflammatory autoimmune diseases such as VKH, suggesting that this monocyte subset could represent a potentially useful cellular therapeutic target in certain inflammatory diseases, especially when patients have strong side effects in response to GC therapy.

## Data Availability Statement

The datasets presented in this study can be found in online repositories. The names of the repository/repositories and accession number(s) can be found below: Gene Expression Omnibus repository (https://www.ncbi.nlm.nih.gov/geo/query/acc.cgi?acc=GSE166663) with the Series number GSE166663.

## Ethics Statement

The studies involving human participants were reviewed and approved by Ethics Committee of Wuhan Aier Eye Hospital (Clinical Ethical Approval No. 2019IRBKY04). The patients/participants provided their written informed consent to participate in this study.

## Author Contributions

YH, ZL, HJ, and JHa designed the study and wrote the manuscript. YZ, LZ, JW, JY, MH, TX, JHe, SW, CY, and SP collected the peripheral blood samples and provided clinical data. LY and HJ performed the experiments and analyzed the data. All authors critically reviewed the manuscript and made key contributions to the analysis and interpretations of the results. All authors contributed to the article and approved the submitted version.

## Funding

This work was supported by the National Natural Science Foundation of China (2017NSFC81670825 to YH), Technology Innovation Guidance Program of Hunan Province (2017SK50902 to ZL) and Science Research Foundation of Aier Eye Hospital Group (AFM1713D1 to ZL).

## Conflict of Interest

The authors declare that the research was conducted in the absence of any commercial or financial relationships that could be construed as a potential conflict of interest.
