# Corrigendum: Immune Phenotyping of Patients With Acute Vogt-Koyanagi-Harada Syndrome Before and After Glucocorticoids Therapy

**DOI:** 10.3389/fimmu.2021.731824

**Published:** 2021-07-16

**Authors:** Han Jiang, Zhaohui Li, Long Yu, Ying Zhang, Li Zhou, Jianhua Wu, Jing Yuan, Mengyao Han, Tao Xu, Junwen He, Shan Wang, Chengfeng Yu, Sha Pan, Min Wu, Hangyu Liu, Haihong Zeng, Zhu Song, Qiangqiang Wang, Shen Qu, Junwei Zhang, Yafei Huang, Junyan Han

**Affiliations:** ^1^ Department of Immunology, School of Basic Medicine, Tongji Medical College, Huazhong University of Science and Technology, Wuhan, China; ^2^ Retinal and Vitreous Diseases Department of Wuhan Aier Eye Hospital, Wuhan University, Wuhan, China; ^3^ Department of Pathogen Biology, School of Basic Medicine, Tongji Medical College, Huazhong University of Science and Technology, Wuhan, China; ^4^ Ophthalmic Imaging Department of Wuhan Aier Eye Hospital, Wuhan University, Wuhan, China; ^5^ Cataract Department of Wuhan Aier Eye Hospital, Wuhan University, Wuhan, China

**Keywords:** immunopathogenesis, lymphocyte subsets, monocytes, VKH, autoimmunity, cytokine

In the original article, there was a mistake in **Figure 3** as published. For the left panel of **Figure 3B**, the label of the y-axis should be ‘% of Tc’ instead of ‘% of Th’**.** The corrected **Figure 3** appears below.

**Figure 3:**
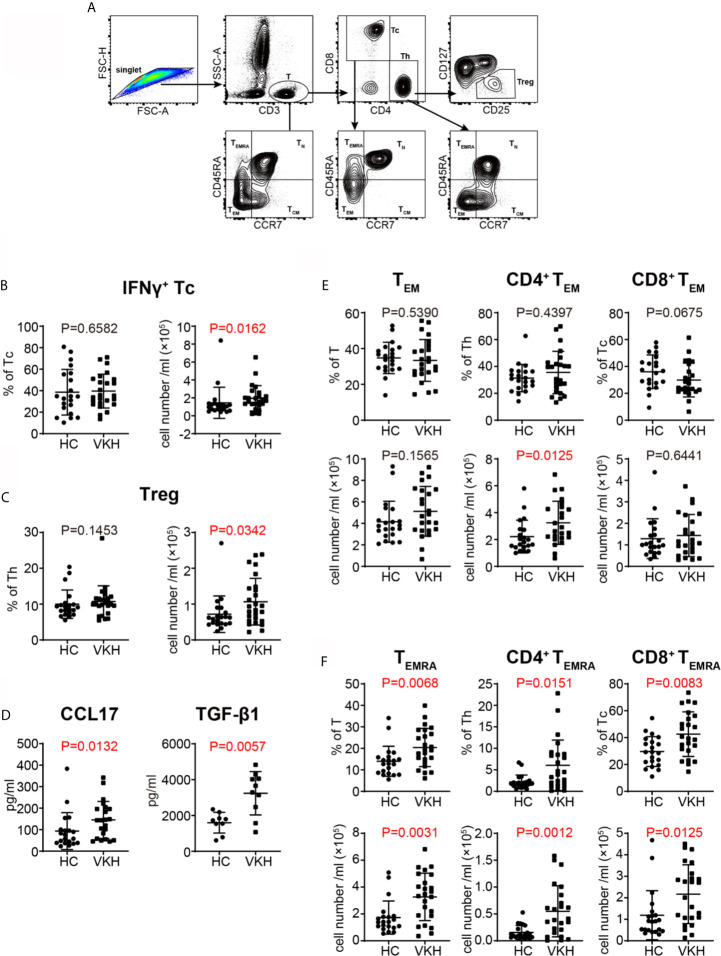
Comparison of T cell subsets defined by cytokine profile and differential status between VKH patients and healthy controls. **(A)** Gating strategy used for T cell classification. **(B)** The absolute number and proportion of IFN-γ+ CD8+ T cell. **(C)** The absolute number and proportion of Treg. **(D)** plasma levels of CCL17 and TGF-β1 (pg/ml) of HCs and VKH patients. **(E)** The absolute number and proportion of TEM in total T cells, CD4+ T and CD8+ T cells in HCs and VKH patients. **(F)** The absolute number and proportion of TEMRA in total T cells, CD4+ T and CD8+ T in HCs and VKH patients. Statistical analysis was performed using Mann–Whitney test. Treg, regulatory T cells; TN, naïve T cells; TCM, central memory T cells; TEM, effector memory T cells; TEMRA, CD45RA+ effector memory T cells.

In the original article, there was another error. In the Results section, ‘serum IL-10 levels’ should be changed to ‘plasma IL-10 levels’.

A correction has been made to Results, GC Treatment Affects the Distribution of Monocyte Subsets in VKH Patients:

**‘**After GC treatment, the proportions of the three monocyte subsets defined by the expression of CD14 and CD16 were significantly altered (**Figure 8A**). Both the proportion and absolute number of CD14^++^CD16^−^ classical subset were increased (**Figure 8B**), whereas the proportion of CD14^++^CD16^+^ intermediate subset, and the proportion and absolute number of CD14^+^CD16^+^ non-classical subset were decreased (**Figures 8C, D**). Interestingly, the newly defined CD14^+^CD56^+^ monocyte subset were significantly increased after GC treatment in terms of both relative frequency and absolute number (**Figure 8E**), indicating that this monocyte subset might play a role in the remission of VKH. Additionally, we examined the concentration of cytokines related to monocyte in the plasma of VKH patients before and after GC treatment, and found that CCL2, a chemokine with the potential to recruit monocyte and T cell to the sites of inflammation induced by either tissue injury or infection (39), were also decreased after GC treatment. In addition, IL-10, a regulatory cytokine secreted by monocyte and Treg, was decreased after GC treatment as well, however, plasma IL-10 levels were extremely low in both groups and the biological meaning of this difference is questionable (**Figures 8F, G**). Therefore, whether IL-10 and CCL2 are involved in the function of CD14^+^CD56^+^ monocytes in GC treatment of VKH patients warrants further investigations.’

The authors apologize for these errors and state that they do not change the scientific conclusions of the article in any way. The original article has been updated.

