# Supplementary material for: Immune Phenotyping of Patients With Acute Vogt-Koyanagi-Harada Syndrome Before and After Glucocorticoids Therapy
Source: Front Immunol. 2021 Apr 28;12:659150. doi: 10.3389/fimmu.2021.659150 (PMC8113950; doi:10.3389/fimmu.2021.659150)
Supplement: Supplementary file 1 [file DataSheet_1.zip › supplementary Data/Supplementary Figure legends .docx]

**FIGURE S1** Comparison of white blood cells in peripheral blood between VKH patients and healthy controls. The absolute number and proportion of granulocytes (**A**), lymphocytes (**B**) and CD3^+^CD56^+^ T cells (**C**). Statistical analysis was performed using Mann–Whitney test.

**FIGURE S2** Comparison of activated immune cells between VKH patients and healthy controls. The absolute number and proportion of NKp46^+^ NK cells (**A**) and NKG2D^+^ NK cells (**B**). Statistical analysis was performed using Mann–Whitney test.

**FIGURE S3** Comparison of the activation and differentiation status between VKH patients and healthy controls. (**A-E**) The number and proportion of Th1, Th2, Th17, IL-4^+^CD8^+^ T, IL-17A^+^CD8^+^ T cells. (**F**) The number and proportion of T_N_ in total T cells, CD4^+^ and CD8^+^ T cells. (**G**) The number and proportion of T_CM_ in total T cells, CD4^+^ and CD8^+^ T cells. (**H**) The T_CM_/T_EM_ ratio of total T cells, CD4^+^ and CD8^+^ T cells. Statistical analysis was performed using Mann–Whitney test.

**FIGURE S4** Changes of B cell subsets and intermediate monocytes in VKH patients compared with healthy controls. (**A-C**) The number and proportion of naïve B cells, switched memory B cells, and double negative B cells. (**D**) The number and proportion of intermediate monocytes. Statistical analysis was performed using Mann–Whitney test.

**FIGURE S5** Changes of white blood cells in 16 VKH patients before and after GC treatment. The number and proportion of B cells (**A**) and CD3^+^CD56^+^ T cells (**B**). Statistical analysis was performed using Wilcoxon test.

**FIGURE S6** Changes of activated NK cells and T cells in 16 VKH patients before and after GC treatment. The number and proportion of NKp46^+^ NK cells (**A**), HLA-DR^+^ T cells (**B**), HLA-DR^+^ Tc cells (**C**), HLA-DR^+^ Th cells (**D**), NKp46^+^ T cells (**E**), NKG2D^+^ T cells (**F**), and Tregs (**G**). Statistical analysis was performed using Wilcoxon test.

**FIGURE S7** Changes of T cell polarization status in 16 VKH patients before and after GC treatment. The number and proportion of Th1 (**A**), Th2 (**B**), Th17 (**C**), IFN-γ^+^ Tc (**D**), IL-4^+^ Tc (**E**), IL-17A^+^ Tc (**F**). Statistical analysis was performed using Wilcoxon test.

**FIGURE S8** Changes of T cell differentiation status in 16 VKH patients before and after GC treatment. (**A**) Flow cytometry analysis of T cell differentiation. (**B**) The T_CM_/T_EM_ ratio in T cell and CD4^+^ T cell compartments, respectively. The number and proportion of T_N_ (**C**), T_CM_ (**D**), T_EM_ (**E**), T_EMRA_ (**F**) in total T cells, CD4^+^ and CD8^+^ T cell compartments. Statistical analysis was performed using Wilcoxon test.

**FIGURE S9** Gene expression in the peripheral blood from seven VKH patients before and after GC treatment for one week. Functional enrichment analysis of genes that were up-regulated in the comparison of VKH patients before and after GC treatment using the KEGG (**A**) and GO enrichment (**B**), (**C**) The relative expression of *NOS3* genes as determined by qRT-PCR. (for each group, n = 3). Statistical analysis was performed using one-way ANOVA.

**FIGURE S10** Changes of B cell subsets in VKH patients before and after GC treatment. (**A**) Flow cytometry analysis of B cells. (**B**) The number and proportion of naïve B cell, double negative B cell, switched B cell and unswitched B cell. Statistical analysis was performed using Wilcoxon test.

**FIGURE S11** The Fluorescence Minus One (FMO) control of CD56 (**A**) and CD16 (**B**), Flow cytometry analysis of granulocyte (**C**).

**FIGURE S12** The FMO control of NKG2D (**A**), NKp46 (**B**) and HLA-DR (C).
