# Supplementary figures and images for: Immune Phenotyping of Patients With Acute Vogt-Koyanagi-Harada Syndrome Before and After Glucocorticoids Therapy
Source: Front Immunol. 2021 Apr 28;12:659150. doi: 10.3389/fimmu.2021.659150 (PMC8113950; doi:10.3389/fimmu.2021.659150)

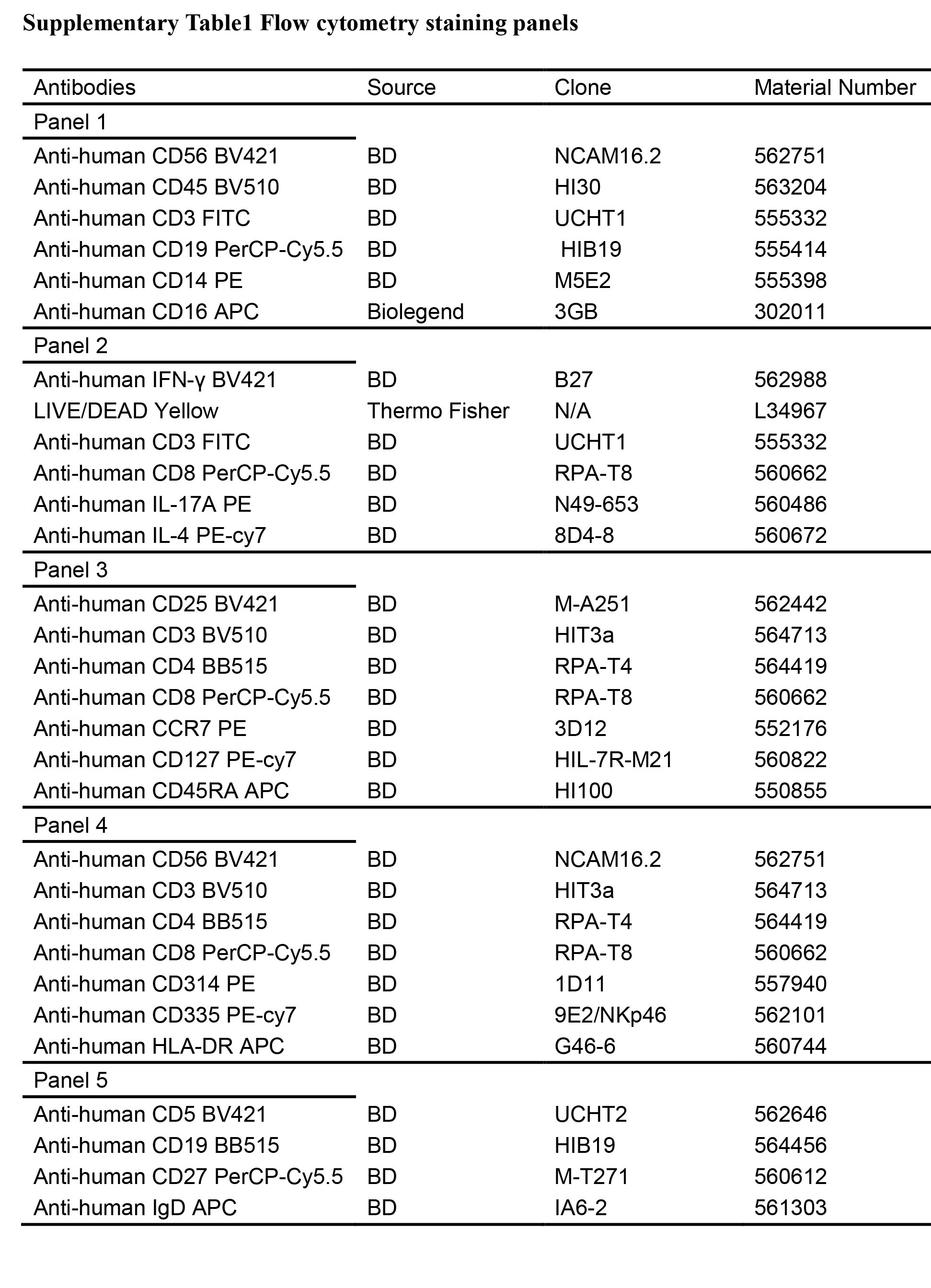

Supplement: Supplementary file 1 [file DataSheet_1.zip › supplementary Data/S table 1.tif]

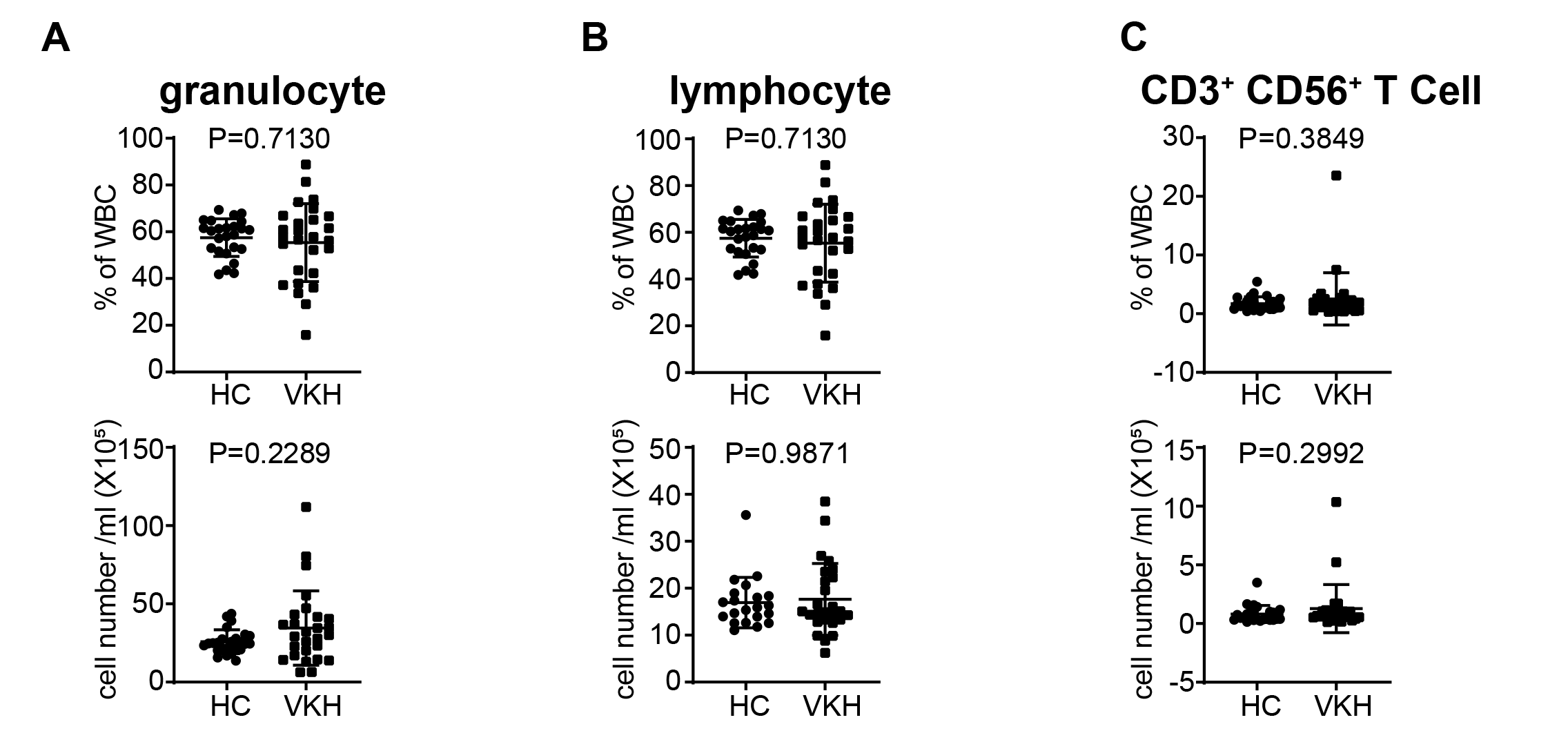

Supplement: Supplementary file 1 [file DataSheet_1.zip › supplementary Data/S1.tif]

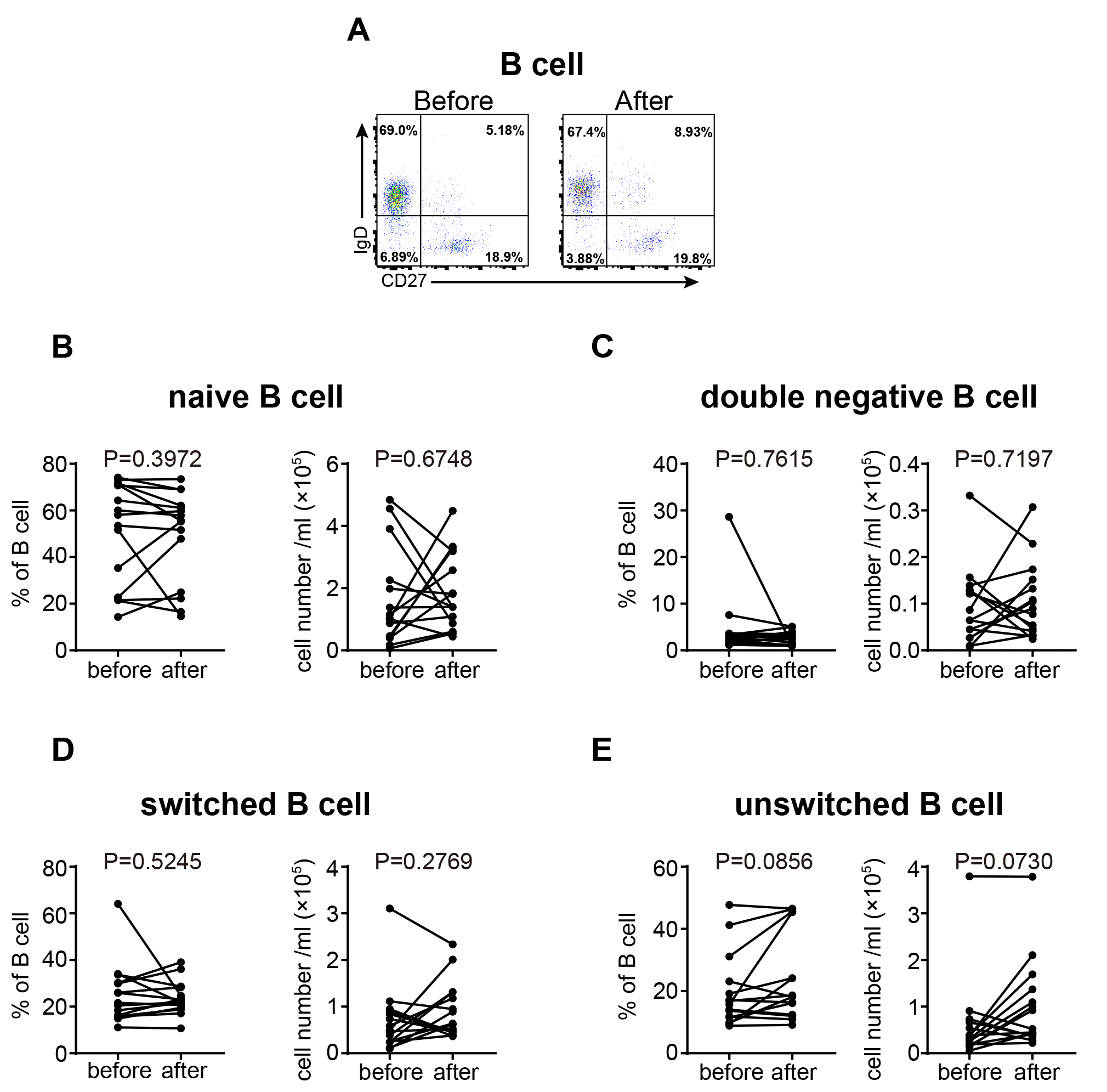

Supplement: Supplementary file 1 [file DataSheet_1.zip › supplementary Data/S10.tif]

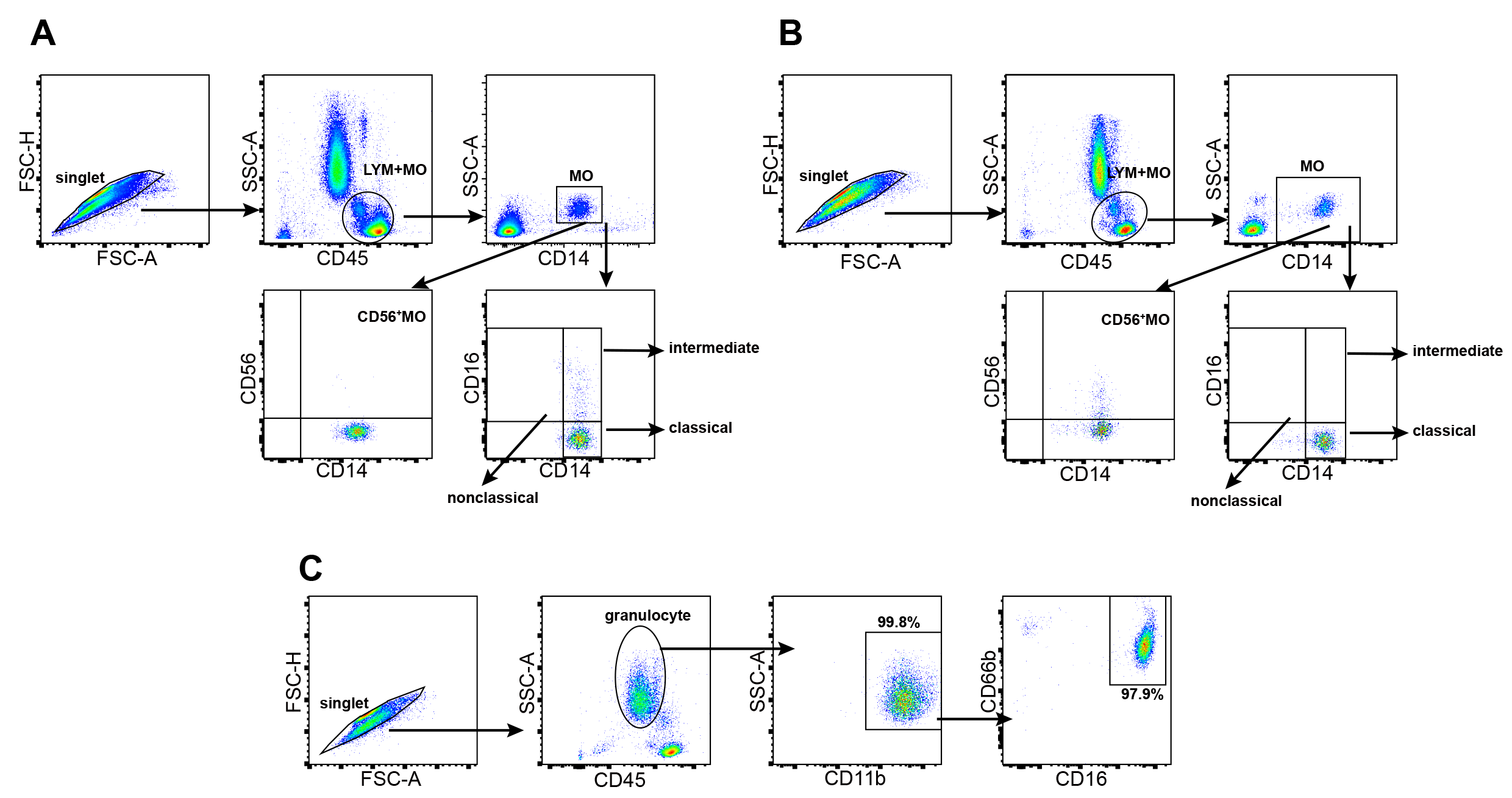

Supplement: Supplementary file 1 [file DataSheet_1.zip › supplementary Data/S11.tif]

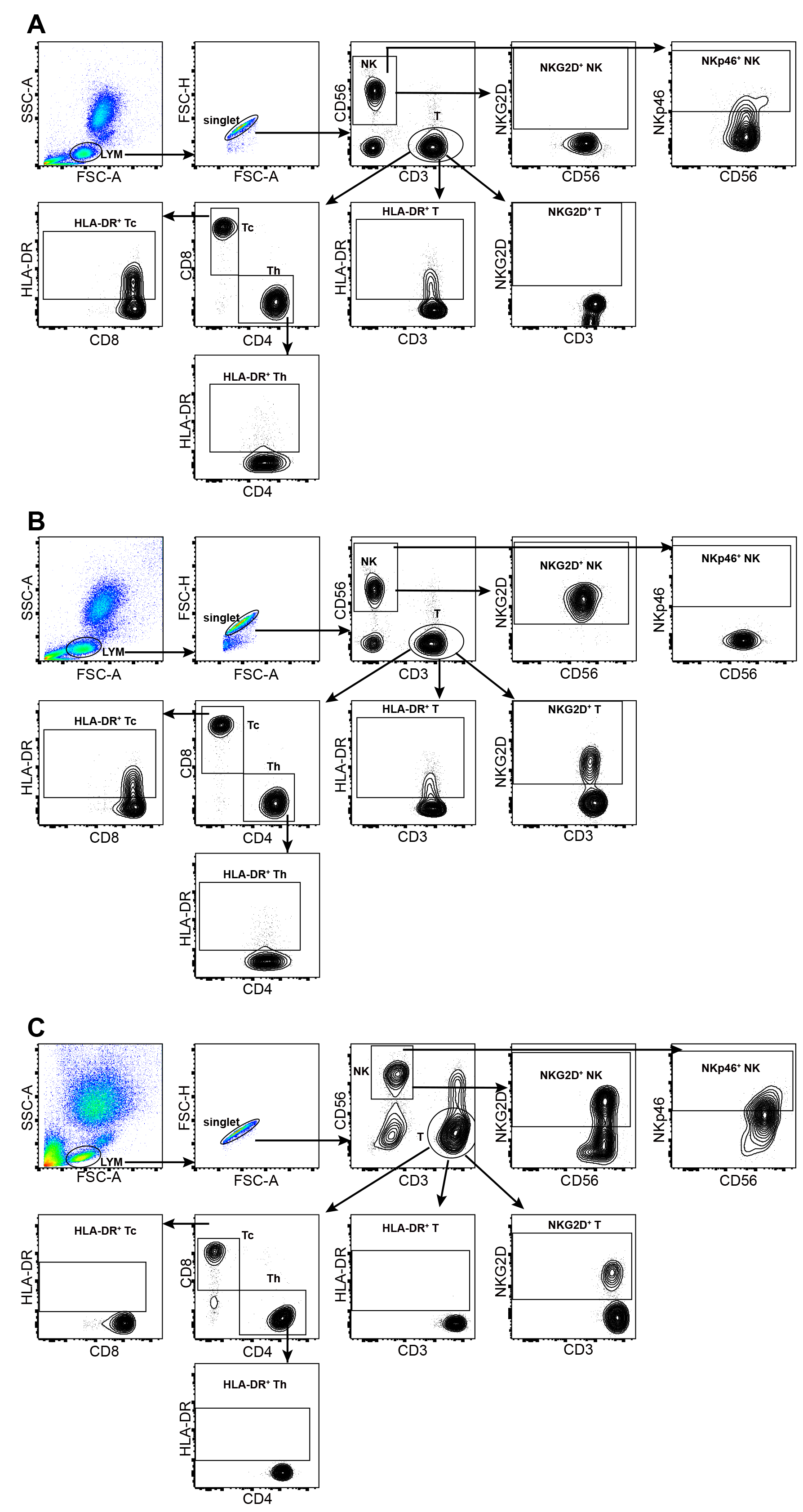

Supplement: Supplementary file 1 [file DataSheet_1.zip › supplementary Data/S12.tif]

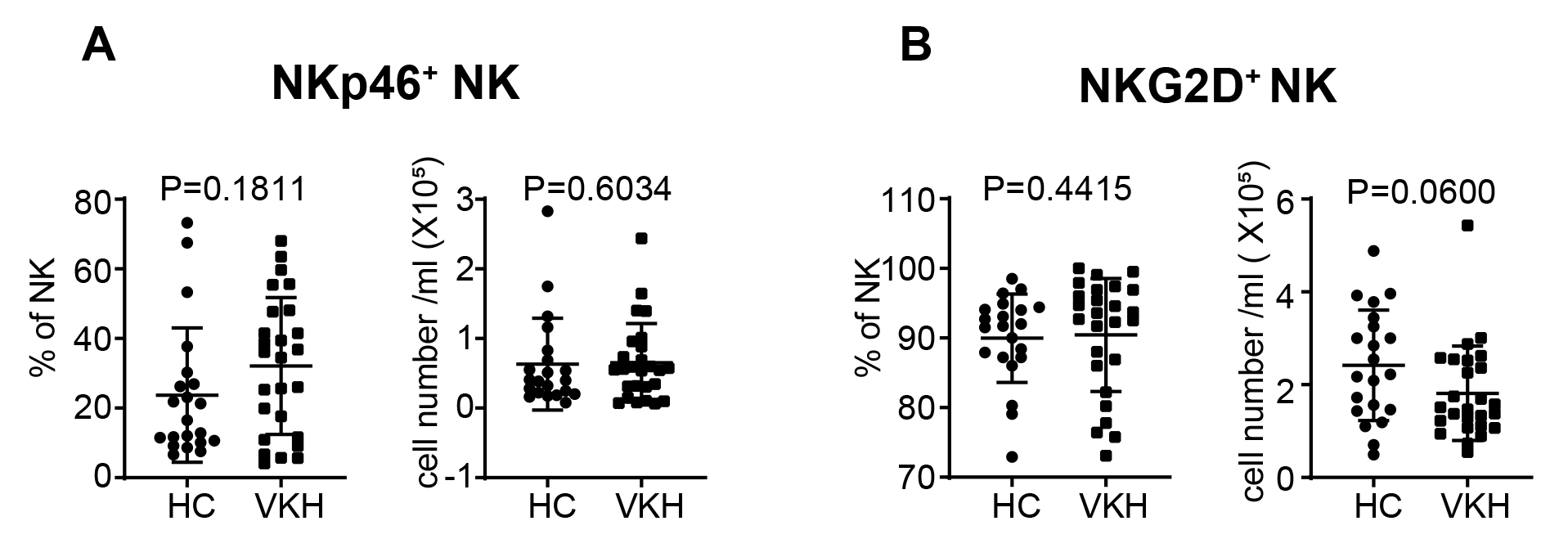

Supplement: Supplementary file 1 [file DataSheet_1.zip › supplementary Data/S2.tif]

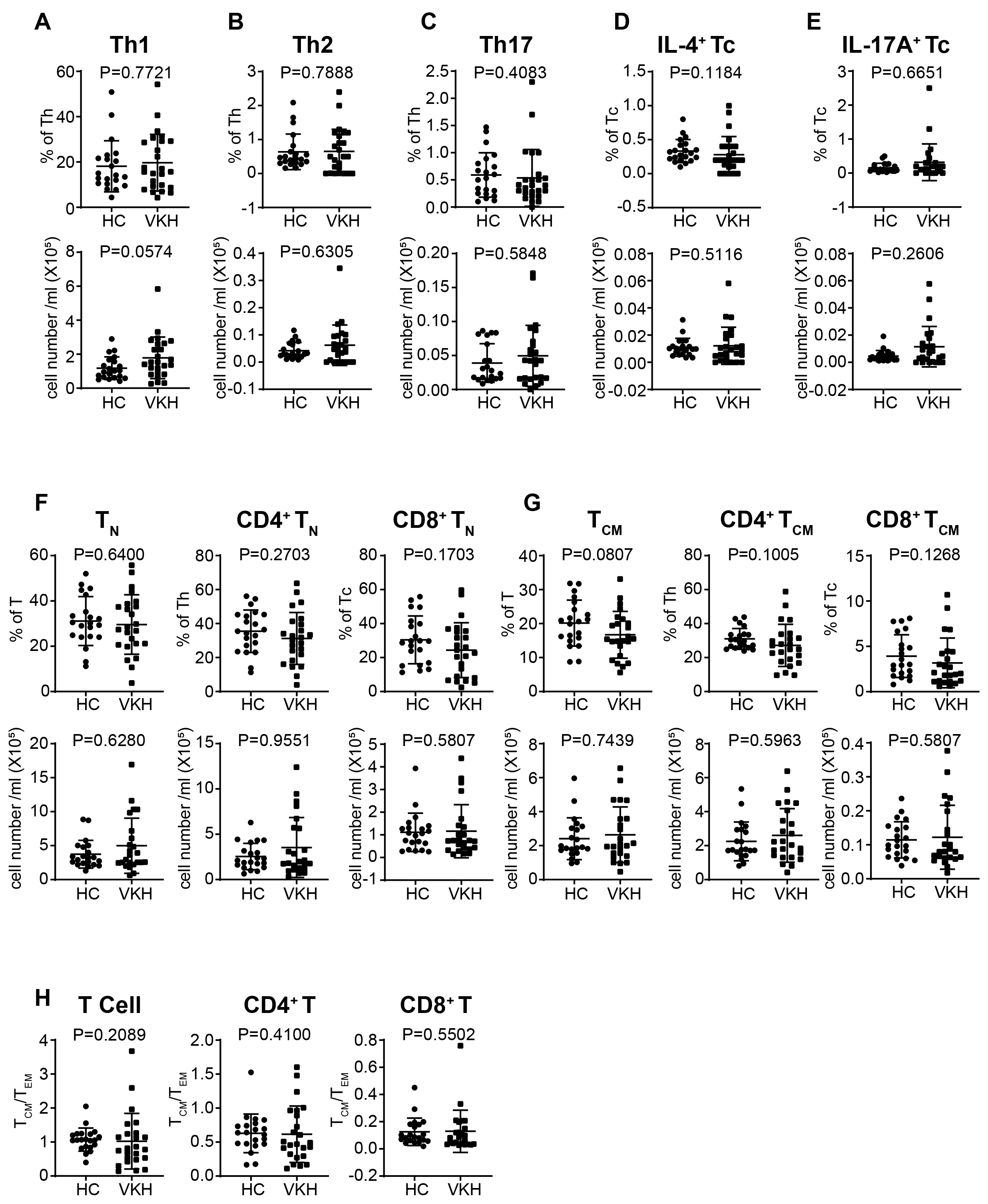

Supplement: Supplementary file 1 [file DataSheet_1.zip › supplementary Data/S3.tif]

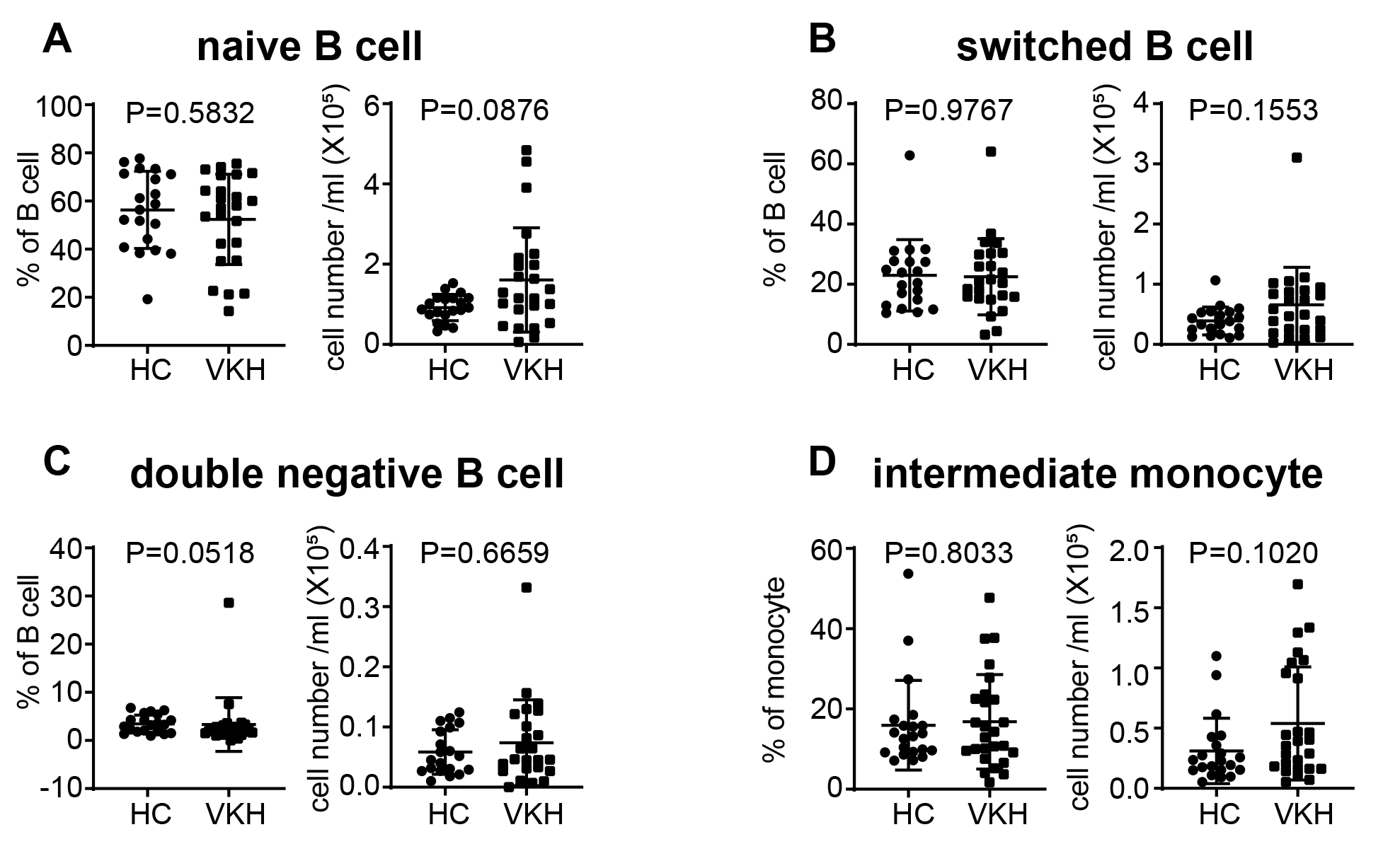

Supplement: Supplementary file 1 [file DataSheet_1.zip › supplementary Data/S4.tif]

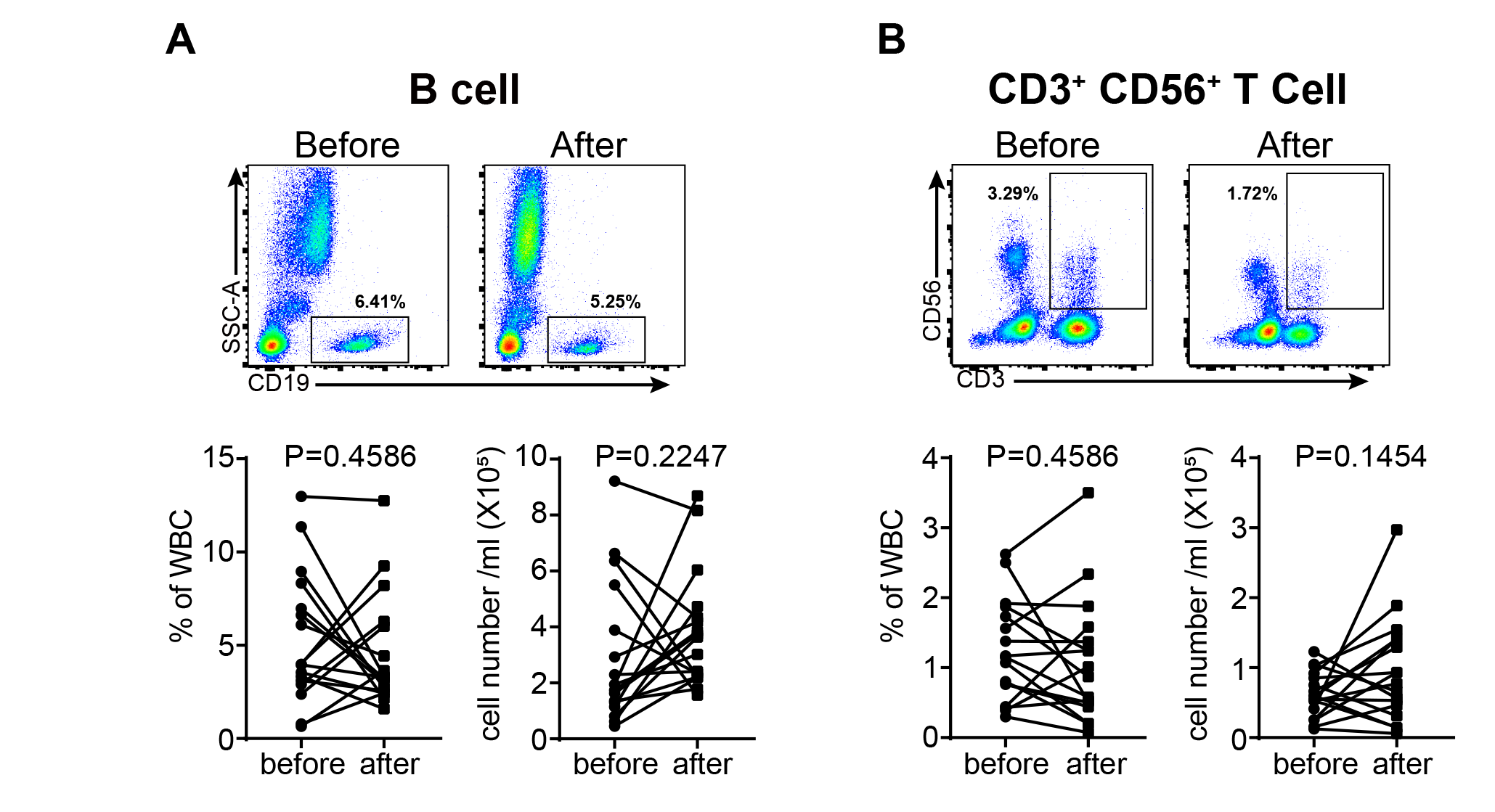

Supplement: Supplementary file 1 [file DataSheet_1.zip › supplementary Data/S5.tif]

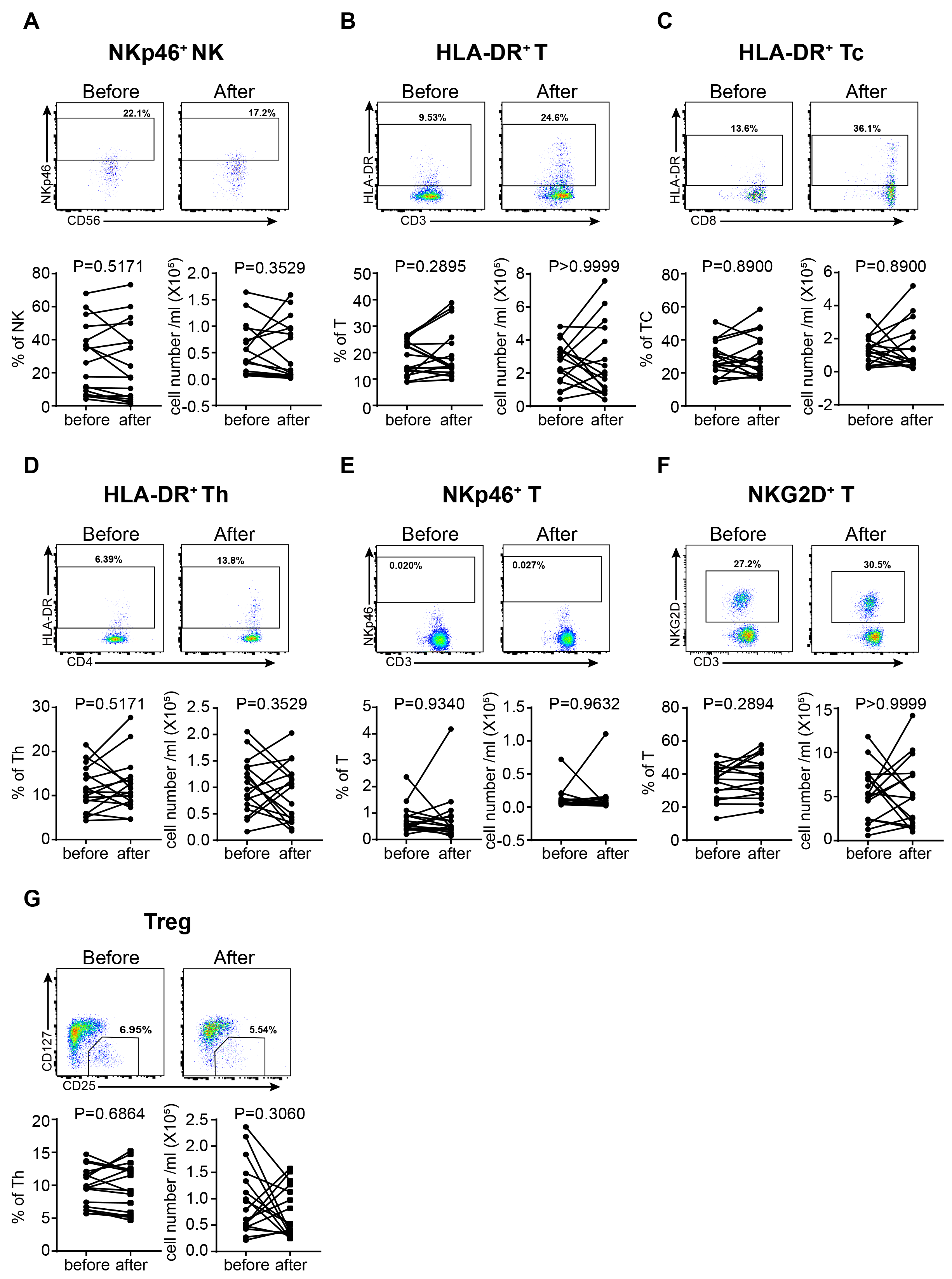

Supplement: Supplementary file 1 [file DataSheet_1.zip › supplementary Data/S6.tif]

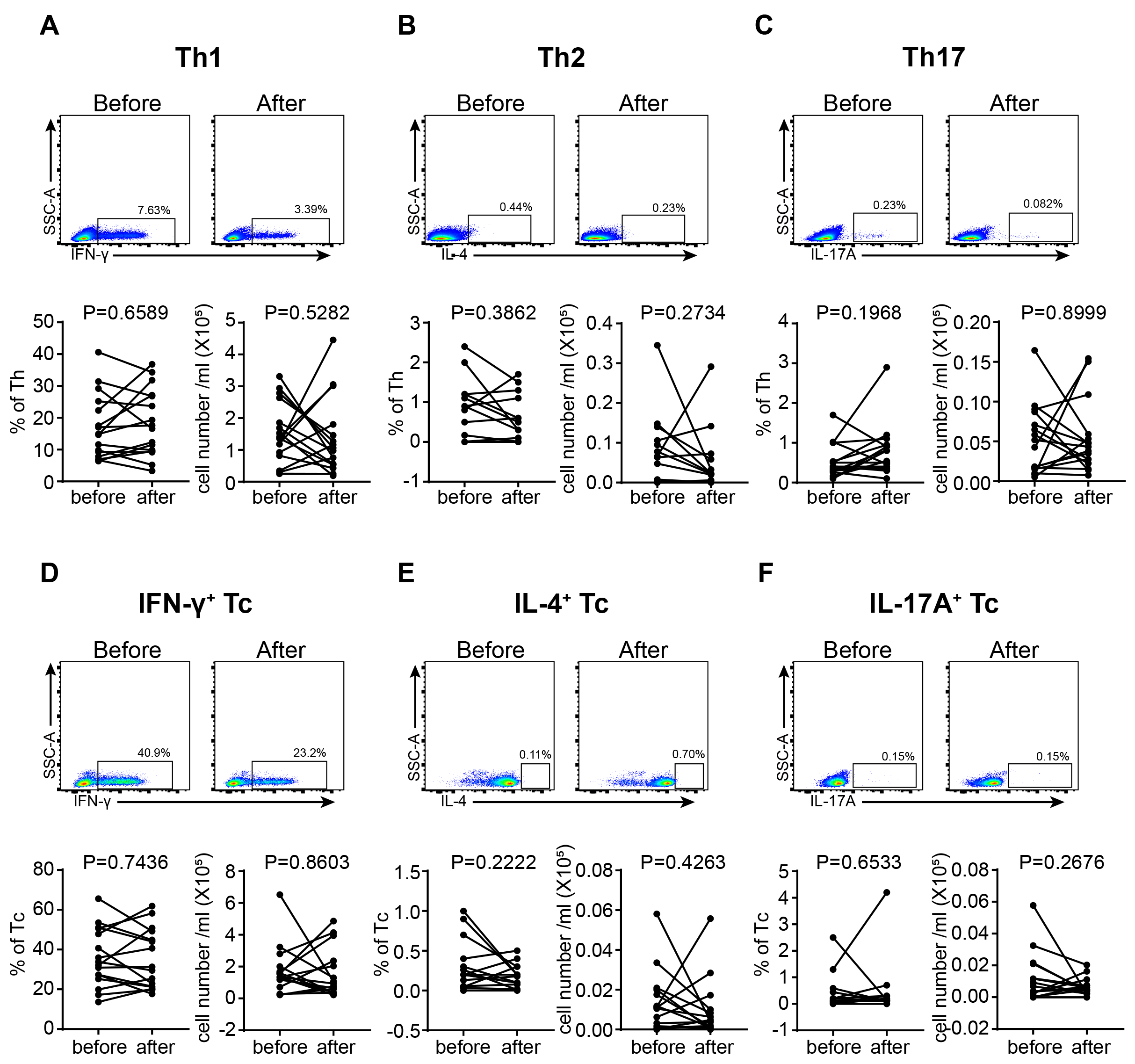

Supplement: Supplementary file 1 [file DataSheet_1.zip › supplementary Data/S7.tif]

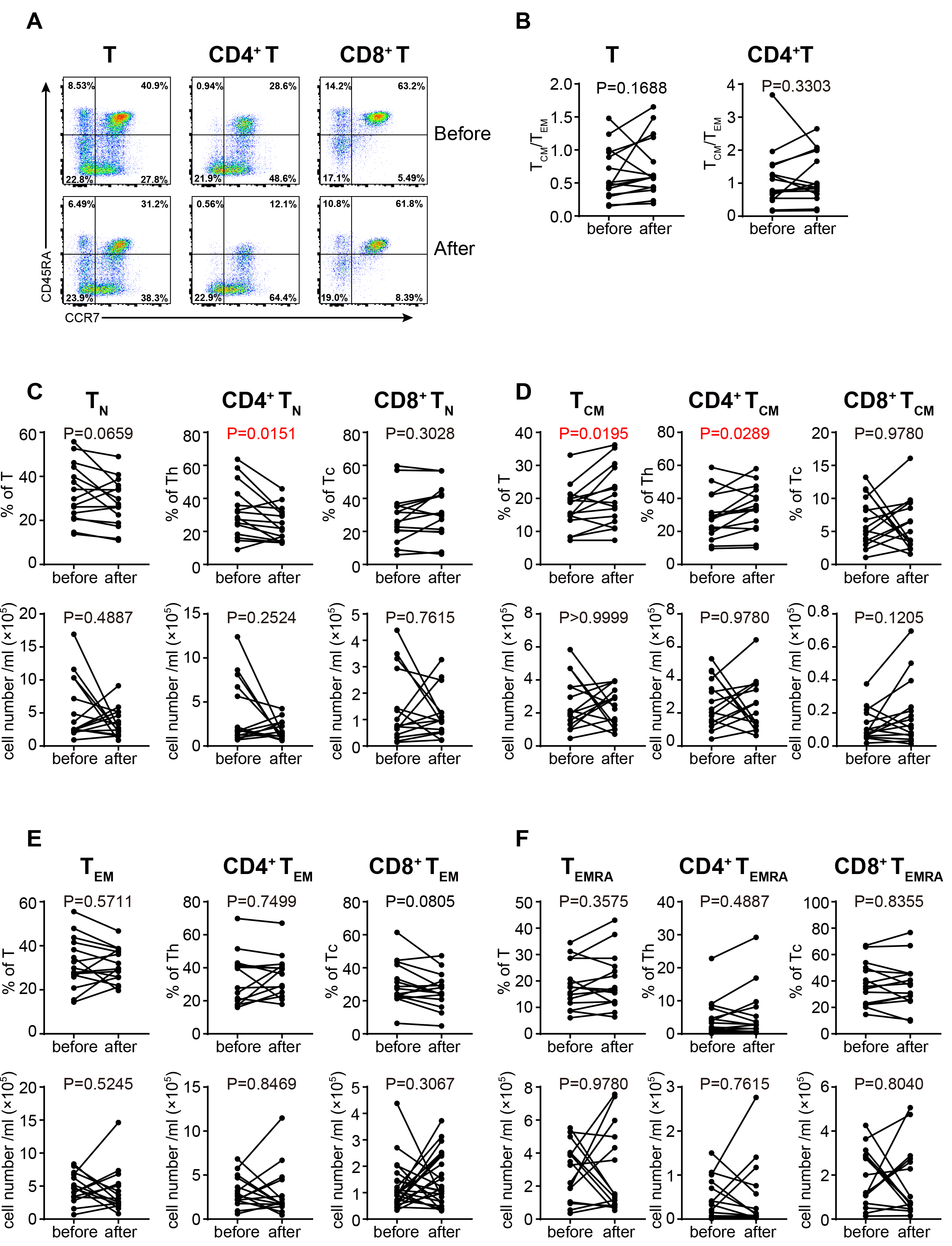

Supplement: Supplementary file 1 [file DataSheet_1.zip › supplementary Data/S8.tif]

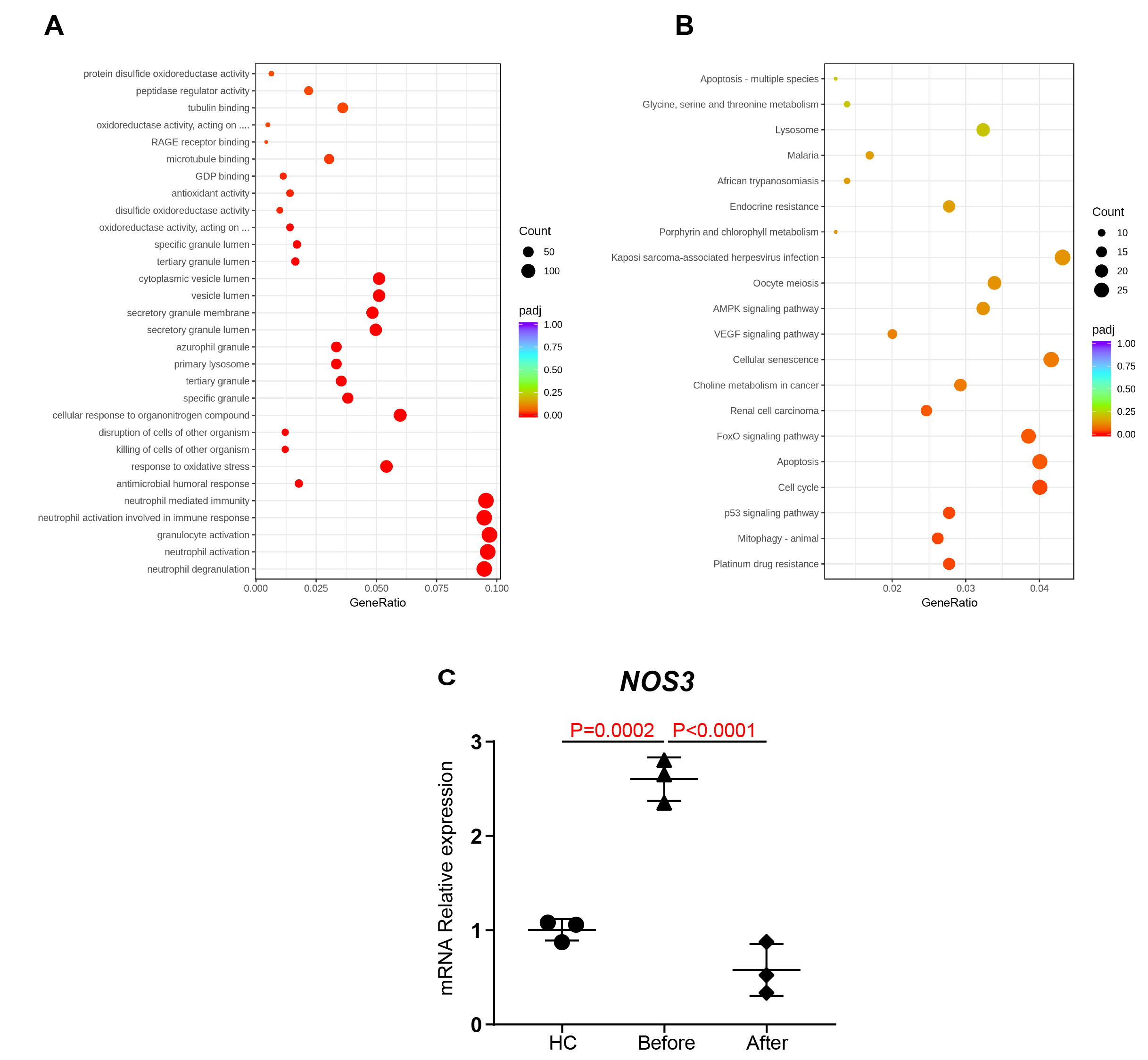

Supplement: Supplementary file 1 [file DataSheet_1.zip › supplementary Data/S9.tif]
